# Endogenous antigens shape the transcriptome and TCR repertoire in an autoimmune arthritis model

**DOI:** 10.1172/JCI174647

**Published:** 2024-11-26

**Authors:** Elizabeth E. McCarthy, Steven Yu, Noah Perlmutter, Yuka Nakao, Ryota Naito, Charles Lin, Vivienne Riekher, Joe DeRisi, Chun Jimmie Ye, Arthur Weiss, Judith F. Ashouri

**Affiliations:** 1 Rosalind Russell and Ephraim P. Engleman Rheumatology Research Center, Department of Medicine,; 2 Department of Epidemiology and Biostatistics, and; 3 Institute for Human Genetics, UCSF, San Francisco, California, USA.; 4 Stanford University, Palo Alto, California, USA.; 5 Friedrich-Alexander University of Erlangen-Nuremburg, Bavaria, Germany.; 6 Department of Biochemistry and Biophysics, UCSF, San Francisco, California, USA.; 7 Chan Zuckerberg Biohub, San Francisco, California, USA.; 8 Bakar Computational Health Sciences Institute, UCSF, San Francisco, California, USA.

**Keywords:** Autoimmunity, Immunology, Autoimmune diseases, T cell receptor, Tolerance

## Abstract

The development of pathogenic autoreactive CD4^+^ T cells, particularly in the context of impaired signaling, remains poorly understood. Unraveling how defective signaling pathways contribute to their activation and persistence is crucial for identifying new therapeutic targets. We performed bulk and single-cell RNA-Seq (scRNA-Seq) and single-cell T cell receptor sequencing (scTCR-Seq) to profile a highly arthritogenic subset of naive CD4^+^ T cells from BALB/c-Zap70*W163C (SKG) mice, which develop CD4^+^ T cell–mediated autoimmune arthritis driven by a hypomorphic mutation in Zap70 — a key TCR signaling kinase. Despite impaired signaling, these cells exhibited heightened expression of T cell activation and cytokine signaling genes but diminished expression of a subset of tolerogenic markers (*Izumo1r*, *Tnfrsf9*, *Cd5*, *S100a11*) compared with WT cells. The arthritogenic cells showed an enrichment for TCR variable β (Vβ) chains targeting superantigens (Sags) from the endogenous mouse mammary tumor virus (MMTV) but exhibited diminished induction of tolerogenic markers following peripheral antigen encounter, contrasting with the robust induction of the negative regulators seen in WT cells. In arthritic joints, cells expressing Sag-reactive Vβs expanded alongside detectable MMTV proviruses. Antiretroviral treatment and Sag-reactive T cell depletion curtailed SKG arthritis, suggesting that endogenous retroviruses disrupted peripheral tolerance and promoted the activation and differentiation of autoreactive CD4^+^ T cells into pathogenic effector cells.

## Abstract 2

Self-reactive naïve CD4+ T cells with impaired TCR signaling escape negative selection and exhibit an autonomous peripheral tolerance defect. Combined with chronic antigen exposure, this promotes arthritogenic T cell responses.

## Graphical abstract

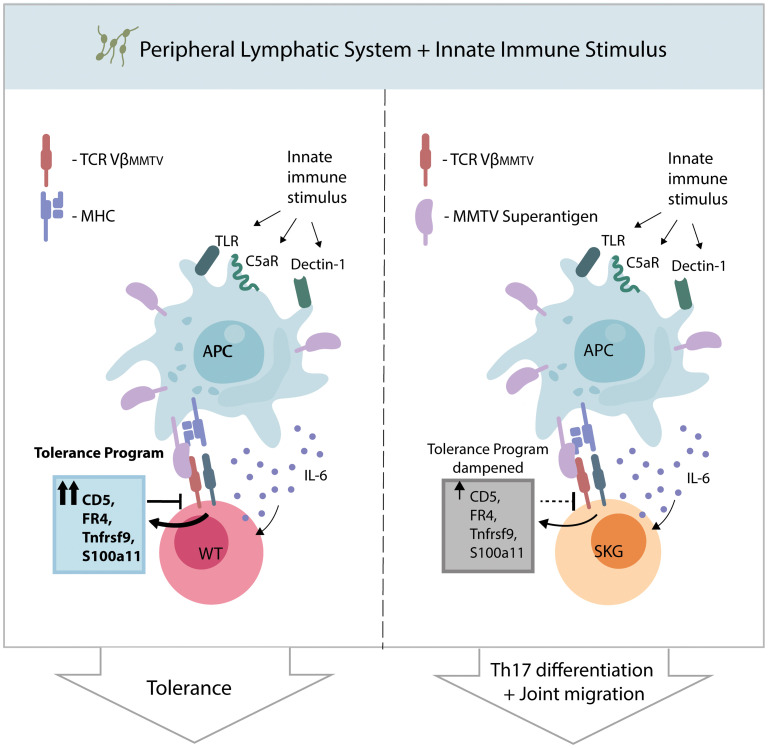

## Introduction

The connection between immunodeficiency and autoimmunity has long intrigued clinical immunologists. This dynamic is particularly evident in patients with primary immunodeficiency, a quarter of whom develop autoimmune conditions, such as autoimmune arthritis (1–3). Some autoimmune diseases are believed to arise from the activation of conventional CD4^+^ T cells that recognize self-antigens (4).

In certain T cell–mediated autoimmune diseases like rheumatoid arthritis (RA), a paradox arises: CD4^+^ T cells can adopt a pathogenic effector state despite impaired T cell receptor (TCR) signaling (5–11). How these T cells evade tolerance to drive disease remains unclear. Self-tolerance is maintained through T cell–intrinsic mechanisms during thymic development, primarily through negative selection, and in the periphery via functional unresponsiveness or “anergy.” Dysregulated T cell signaling is implicated in the pathogenesis of T cell–mediated autoimmune diseases, as seen in both humans and murine models. The BALB/c-Zap70*W163C (SKG) mouse model of autoimmune arthritis offers valuable insights into the mechanisms driving these complex interactions.

The SKG mouse, derived from the BALB/c strain, serves as a powerful tool for studying how impaired immunity leads to tolerance defects and autoimmune arthritis. A hypomorphic mutation in *Zap70*, a key tyrosine kinase in TCR signaling, disrupts thymocyte negative selection, allowing self-reactive CD4^+^ T cells to escape into the periphery (12–15). When exposed to environmental triggers, these dormant arthritogenic CD4^+^ T cells become activated, causing erosive inflammatory arthritis resembling RA (12, 16) and sharing features with spondyloarthritis (17). SKG CD4^+^ T cells are required to cause arthritis (12), and even naive CD4^+^ (CD4^+^_naive_) T cells transferred into immunodeficient hosts trigger disease (13). However, the mechanisms driving their differentiation into pathogenic effector cells, despite impaired TCR signaling, remain unclear.

To investigate this, we used the SKGNur mouse, combining the SKG model with the Nur77-EGFP TCR signaling reporter (13). In this model, GFP is expressed under the control of *Nr4a1*, which encodes NUR77, a TCR signaling marker. Antigen, but not inflammation, triggers GFP expression, allowing us to track antigen-activated T cells (18, 19) during disease progression. Our previous studies revealed that high Nur77-EGFP expression marks self-reactive CD4^+^_naive_ T cells with heightened IL-6 sensitivity, making them more arthritogenic (Supplemental Figure 1A; supplemental material available online with this article; https://doi.org/10.1172/JCI174647DS1) (13). Chronic antigen exposure in peripheral SKG CD4^+^ T cells emerges as a potential catalyst, downregulating SOCS3 expression — a key regulator of IL-6 signaling — an observation mirrored in patients with RA (13). We hypothesize that additional immune regulators may be dysregulated in SKG CD4^+^ T cells, heightening their susceptibility to peripheral tolerance breakdown.

To test this, we analyzed the transcriptome and TCR repertoire of SKG CD4^+^_naive_ T cells using bulk and scRNA-Seq. The SKGNur model allowed us to identify and capture arthritogenic (SKGNur GFP^hi^) cells before disease onset, similar to the preclinical phase of autoimmune disease (20). This provided insight into early pathogenic mechanisms and potential targets for tolerance preservation. Within GFP^hi^ CD4^+^_naive_ T cells, a subset with elevated *Nr4a1* expression (T.N4*_Nr4a1_*) exhibited gene programs linked to TCR signaling in response to antigen engagement. However, SKGNur GFP^hi^ T.N4*_Nr4a1_* cells showed impaired induction of a subset of tolerogenic genes compared with WT counterpart cells. TCR-Seq revealed a significant enrichment of variable β (Vβ) chains (FDR < 0.1), validated by protein analysis (*P* < 0.05), in SKG T cells that recognize superantigens (Sags) from mouse mammary tumor virus (MMTV), an endogenous retrovirus (ERV) in BALB/c mice. Dysregulated TCR signaling was evident in these Sag-reactive cells, as was a greater propensity for Th17 differentiation. Furthermore, these Sag-reactive CD4^+^ T cells expanded within arthritic joints of SKG mice, potentially driving arthritis. Treatment with antiretrovirals or depletion of Sag-reactive T cells significantly delayed arthritis onset (*P* < 0.01 and *P* < 0.05 respectively). These results underscore a breakdown in peripheral tolerance among self-reactive T cells that bypass negative selection. Coupled with chronic antigen stimulation, these circumstances set the stage for autoimmune disease. Our findings also reveal a unique role for ERV Sags in driving pathogenic T cell responses that contribute to disease development.

## Results

### Arthritogenic SKG CD4^+^_naive_ T cells display a gene signature of TCR activation.

**Figure 1:**
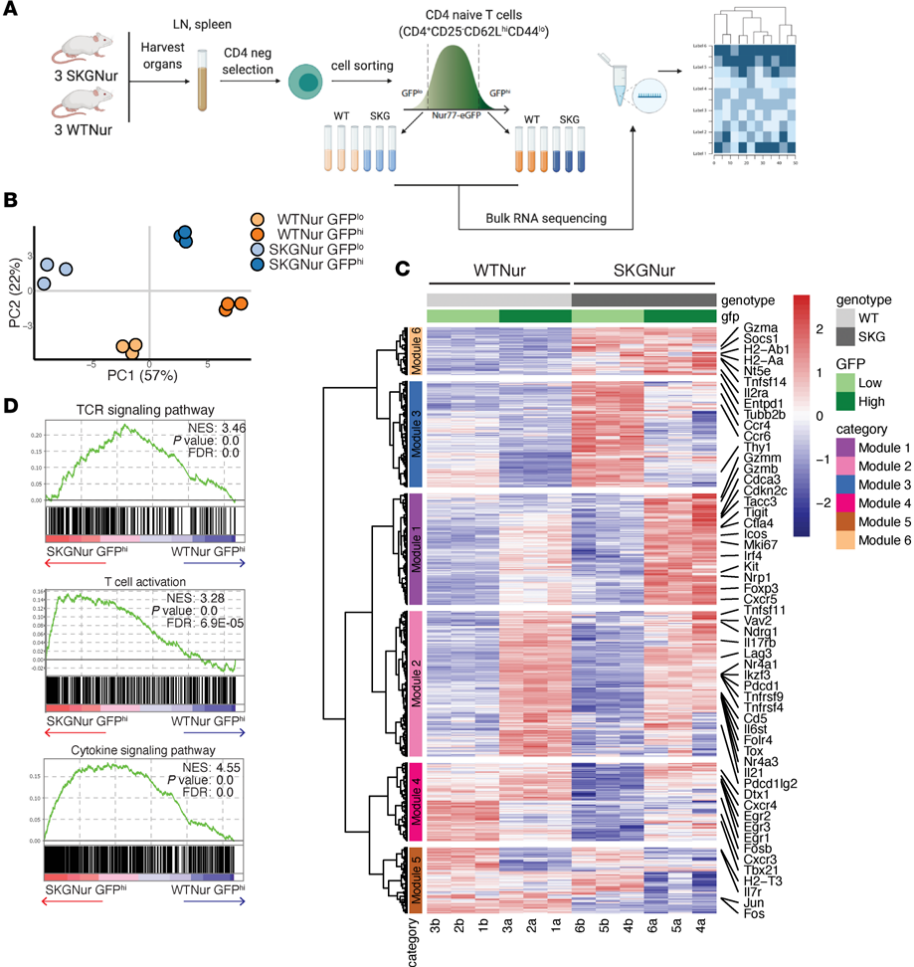
Prearthritic naive SKG T cells demonstrate enhanced T cell activation. (**A**) Experimental schematic of the bulk RNA-Seq analysis. neg, negative. (**B**) PCA based on transcriptomics data from bulk RNA-Seq reveals the distribution of SKGNur and WTNur GFP^lo^ and GFP^hi^ CD4 naive T cell subsets as shown in **A** (*n* = 3 per subgroup). (**C**) Heatmap showing the expression of 991 significantly DEGs [|log_2_(fold change [FC])| >1, adjusted (adj.) *P* < 0.05] from pairwise comparisons for all samples grouped by subgroup. Hierarchical clustering was used to group DEGs into 6 modules (indicated by dendrogram and row annotation color bar on left). (**D**) Enrichment plots of TCR signaling and cytokine pathways from gene set enrichment analysis (GSEA) analysis of all Gene Ontology Biological Process (GOBP) pathways for ranked genes from SKGNur GFP^hi^ and WTNur GFP^hi^ differential expression analysis. NES, normalized enrichment score.

We previously identified arthritogenic CD4^+^_naive_ T cells prior to disease onset using Nur77-EGFP expression in SKG mice (13). To understand the transcriptional profile of these SKGNur GFP^hi^ cells, we performed bulk RNA-Seq on naive (CD62L^hi^CD44^lo^CD25^–^) CD4^+^ T cells with the highest (GFP^hi^) and lowest (GFP^lo^) GFP expression; these cells were obtained from SKGNur and WT (WTNur) mice before arthritis onset (Figure 1A, Supplemental Figure 1B, and Supplemental Data File 1). Principal component analysis (PCA) revealed that all 4 subgroups were transcriptionally distinct (Figure 1B). Hierarchical clustering of the 991 differentially expressed genes (DEGs) between groups identified 6 gene modules (Figure 1C and Supplemental Data File 2). Gene ontology analysis (21) highlighted functional differences between, and in some cases within, these modules (Supplemental Figure 1C). Among the 260 genes with differential expression between WTNur GFP^hi^ and SKGNur GFP^hi^ cells (Supplemental Figure 1, D and E), SKGNur GFP^hi^ cells showed higher expression of cell-cycle genes (*Cdca3*, *Cdk2nc*, *Mki67*, represented in module 1) and genes linked to cytokine signaling (*Socs1*, *Tnfsf14*, *Il2ra*, *H2-Aa*, *H2-Ab1*, represented in module 6) (Figure 1, C and D, Supplemental Figure 1E, and Supplemental Data Files 1 and 3). Interestingly, module 6 genes were highly expressed in SKGNur cells regardless of GFP expression, suggesting a distinct regulatory pathway in these cells (Figure 1C).

Despite their hypomorphic *Zap70* allele and impaired proximal TCR signaling (12, 13, 15), SKGNur GFP^hi^ cells induced both positive (*Egr1*, *Id3*, *Icos*, *Irf4*, *Tnfrsf9*, *Tnfrsf4*, *Myb*) and negative (*Nr4a1*, *Nr4a3*, *Cd5*, *Folr4/Izumo1r*, *Tigit*, *Tox*, *Pdcd1*, *Lag3*, *Ctla4*, *Birc5*, *Nrp1*) regulators of TCR signaling, which were primarily found in modules 1 and 2 (Figure 1C, Supplemental Figure 1E, and Supplemental Data File 2). Paradoxically, SKGNur GFP^hi^ CD4^+^_naive_ T cells had higher expression of activation and signaling pathway genes compared with WTNur GFP^hi^ cells (Figure 1D and Supplemental Data File 3). This likely reflects the chronic endogenous antigen encounter experienced by self-reactive SKG T cells.

### SKG CD4^+^_naive_ T cells that have most recently encountered antigen demonstrate an enhanced TCR signaling program.

**Figure 2:**
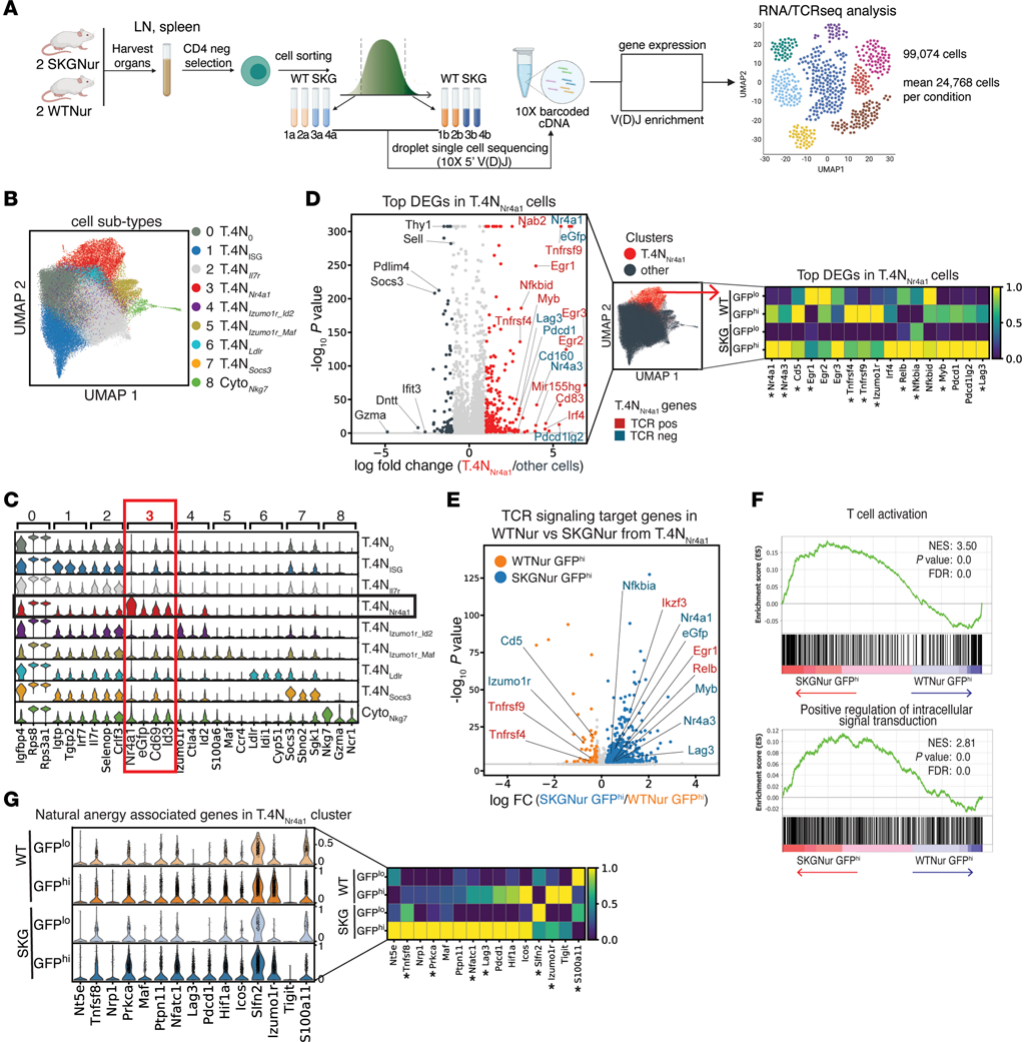
scRNA-Seq reveals heterogeneity in naive CD4^+^ T cells, highlighting a subset defined by TCR signaling genes. (**A**) Experimental design of paired scRNA- and TCR-Seq of sorted GFP^hi^ and GFP^lo^ naive CD4^+^ T cells. V(D)J, variable, diversity, and joining sequences. (**B**) Uniform manifold approximation and projection (UMAP) of 99,074 naive T cells from 8 samples in **A**, colored according to merged clusters. (**C**) Violin plots of log-normalized expression of marker genes for each cluster. Black box highlights the T.4N*_Nr4a1_* cluster; red box highlights genes uniquely expressed in the T.4N*_Nr4a1_* cluster. (**D**) Volcano plot of DEGs in the T.4N*_Nr4a1_* cluster versus all other cells. Dots are colored according to significant overexpression (|log_2_(FC)| >1, adj. *P* < 0.05) in the T.4N*_Nr4a1_* cluster (red) or in other cells (dark gray), or to no significant difference (light gray). Labeled genes are colored according to their role in TCR signaling regulation: positive (red) or negative (blue). Heatmap shows average expression of the labeled genes by subgroup normalized by the standard scale for each gene. (**E**) Volcano plot of DEGs from SKGNur versus WTNur GFP^hi^ cells in the T.4N*_Nr4a1_* cluster. Dots are colored according to significant overexpression (|log_2_(FC)| >0.2, adj. *P* < 0.05) in WTNur (orange) or SKGNur (blue) GFP^hi^ cells or no significant difference in expression between groups (gray). Labeled genes involved in TCR signaling are colored as in **D**. (**F**) Enrichment plots of TCR activation and signaling pathways from GSEA analysis of GOBP pathways for ranked DEGs of SKGNur versus WTNur GFP^hi^ cells from the T.4N*_Nr4a1_* cluster. (**G**) Violin plots show expression of “natural anergy” genes in WTNur, SKGNur GFP^lo^, and GFP^hi^ CD4^+^ naive cells from the T.4N*_Nr4a1_* cluster. Heatmap displays average gene expression by subgroup. Both are normalized by the standard scale. Asterisks in the heatmap labels in **E** and **G** indicate significant differential gene expression between SKGNur and WTNur GFP^hi^ cells.

The long half-life of EGFP (22) compared with the more dynamic turnover of NUR77 protein and *Nr4a1* transcripts (23–25) (Supplemental Figure 2, A–C) means GFP^hi^ cells probably consist of mixed populations of more recently and less recently stimulated cells. To explore the heterogeneity in these subsets, we performed single-cell RNA-Seq (scRNA-Seq) and single-cell TCR-Seq (scTCR-Seq) on GFP^hi^ and GFP^lo^ CD4^+^_naive_ T cells from SKGNur and WTNur mice (Figure 2A). In our scRNA-Seq dataset, we identified 9 distinct clusters (Figure 2, B and C), which recapitulated our bulk RNA-Seq gene signatures (Figure 1 and Supplemental Data File 4) and demonstrate pronounced heterogeneity within the GFP^hi^ cell population (Supplemental Figure 2, D and E). GFP^hi^ CD4^+^ T cells from both SKGNur and WTNur mice were present across all 9 clusters but were enriched in the T.N4*_Nr4a1_* cluster, which had the highest expression of *Nr4a1* and EGFP compared with GFP^lo^ CD4^+^ T cells by a mean of more than 4-fold (Figure 2C, Supplemental Figure 2, F and G, and Supplemental Data File 5). GFP^hi^ T cells were also enriched in the T.N4*_Izumo1r_Id2_* clusters and, to a lesser extent, in the Cyto*_Nkg7_* clusters (Supplemental Figure 2, D–G).

Given its specificity as a reporter of TCR signaling (22, 26, 27), the high expression of *Nr4a1* in the T.N4*_Nr4a1_* cluster signified that these cells most recently encountered endogenous antigen(s) (28, 29). Indeed, T.N4*_Nr4a1_* cells overwhelmingly expressed genes associated with TCR signal transduction (including *Nr4a1*, *Nr4a3*, *Egr1-3*, *Tnfrsf9*, *Tnfrsf4*, *Ifr4*, *Cd5*, and *Cd69*) (Figure 2D and Supplemental Data Files 4 and 5), which most closely mirrored the TCR signaling genes found in module 2 of our bulk RNA-Seq analysis (Supplemental Figure 2H and Supplemental Data File 2). Similarly, we found that surface expression of OX-40 (*Tnfrsf4*), CD5, and CD69 was significantly higher in naive CD4^+^GFP^hi^ T cells compared with expression in naive CD4^+^ GFP^lo^ T cells in WTNur and SKGNur mice (Supplemental Figure 2I).

Several of the TCR response genes highly expressed in cluster T.N4*_Nr4a1_* have been identified as targets of the LAT/PLCγ/HDAC7 pathway (including *Nr4a1*, *Egr1–3*, *Irf4*) and correlate with tonic signaling strength (28, 29). These TCR signaling signatures in the T.N4*_Nr4a1_* cluster were more highly expressed in SKGNur GFP^hi^ CD4^+^ T cells than in WT GFP^hi^ CD4^+^ T cells (including *Nr4a1*, *Nr4a3*, *Relb*, *Nfkbia*, *Myb*, *Lag3* genes) (Figure 2, E and F, and Supplemental Data Files 3 and 5), in congruence with our bulk RNA-Seq dataset. This suggests that, despite their intrinsic signaling defect and dampened inducible signaling (Supplemental Figure 2J and ref. 13), the SKGNur GFP^hi^ cells in cluster T.N4*_Nr4a1_* had responded more strongly to or encountered more antigen than did WTNur GFP^hi^ cells.

### SKGNur GFP^hi^ T cells have impaired expression of a subset of tolerogenic genes.

We next investigated additional T cell transcriptomic signatures to understand how SKGNur GFP^hi^ CD4^+^_naive_ T cells may evade tolerance and differentiate into pathogenic effector cells. We analyzed the expression of candidate genes linked to tolerance programs (30–36) in cells that recently encountered antigen. We found that GFP^hi^ CD4^+^_naive_ T cells compared with GFP^lo^ cells in cluster T.N4*_Nr4a1_* from both WTNur and SKGNur mice induced genes associated with tolerogenic programs (*Izumo1r*, *Pdcd1*, *Lag3*, *Tox)* and additional “natural anergy” genes identified by ElTanbouly et al., including *Nfatc1*, *Hif1a*, and *Icos* (Figure 2, D and G, Supplemental Figure 3A, and Supplemental Data File 5). This likely indicates the activation of a negative regulatory program in CD4^+^_naive_ T cells in response to persistent TCR signaling (13, 37, 38), a process that is partially driven by NR4A family members, which have been shown to play inhibitory roles in peripheral T cells (24, 35, 39, 40).

While genes associated with tolerogenic programs and TCR signaling are broadly induced in SKGNur and WTNur GFP^hi^ cells, we found that several of these TCR negative regulators were less efficiently induced in SKGNur GFP^hi^ cells than in WTNur GFP^hi^ cells in cluster T.N4*_Nr4a1_* (*Izumo1r*, *S100a11*, *Tnfrsf9*, *Cd5*) (Figure 2, D, E, and G, and Supplemental Figure 3A). The lower expression of *Izumo1r*, which encodes folate receptor 4 (FR4), a specific marker of anergic cells, suggests that SKGNur GFP^hi^ CD4^+^ T cells may perhaps suboptimally induce anergy and/or other tolerogenic programs. In our bulk RNA-Seq analysis, we also observed a lower magnitude of *Izumo1r* — also known as *Folr4* — expression in SKGNur GFP^hi^ cells compared with WTNur GFP^hi^ cells (Supplemental Data File 1). Therefore, in addition to a known loss in central tolerance, SKG mice probably have an independent defect in mechanisms maintaining peripheral tolerance. This defect is likely derived from their impaired proximal TCR signaling capacity and may explain the reduced frequency of anergic peripheral CD4^+^ T cells we previously reported in SKGNur mice (13).

### SKG hyperresponsiveness to IL-6 is preprogrammed transcriptionally.

IL-6 production in SKG mice is indispensable for SKG arthritis development (41, 42). Recognition of major histocompatibility complex–self-peptide complexes stimulate antigen-presenting cells (APCs) to secrete IL-6 (42). We previously found that SKGNur GFP^hi^ T cells were more responsive to IL-6 and more readily produced IL-17 in the most self-reactive T cells, in part due to lower levels of SOCS3 — a critical negative regulator of IL-6 (13) (Supplemental Figure 1A). In our current study, we found that genes associated with IL-6 signaling machinery and the Th17 pathway were uniquely enriched in SKGNur GFP^hi^ T cells compared with WTNur GFP^hi^ T cells (43) in the T.N4*_Nr4a1_* cluster (Supplemental Figure 3B).

SOCS3 is suppressed in CD4^+^_naive_ T cells in response to antigen (44) and in patients with RA (13, 45). Its expression has a strong inverse correlation with murine arthritis severity (46–48) and was one of the genes most strongly suppressed in T.4N*_Nr4a1_* cells in our dataset. Therefore, we examined the expression of additional SOCS family members to determine whether this suppression was unique to *Socs3* (Supplemental Figure 3C). Of the SOCS family members, *Socs3* was specifically reduced in GFP^hi^ cells versus GFP^lo^ cells within the T.N4*_Nr4a1_* cluster (Figure 2D and Supplemental Data File 5). Moreover, we found a striking inverse correlation between the expression of *Nr4a1* and *Socs3* (Supplemental Figure 3D), validating our previous results. This highlights the interdependence between signaling via the TCR and heightened sensitivity to cytokines such as IL-6 (13).

### T.N4_Nr4a1_ cells segregate into 2 distinct TCR signaling subclusters.

**Figure 3:**
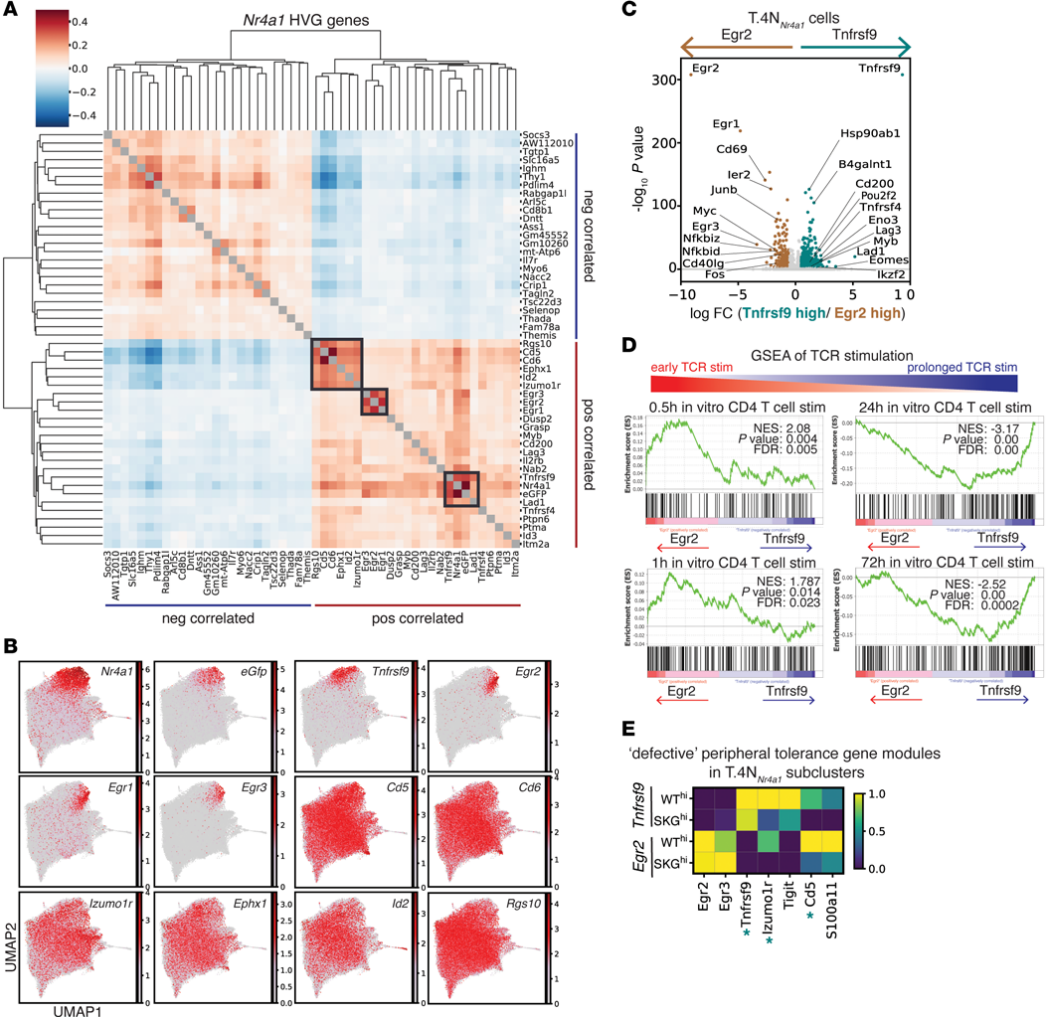
T.N4*_Nr4a1_* cells segregate into 2 distinct TCR signaling modules that segregate acute from chronically antigen-activated T cells. (**A**) Correlation matrix shows hierarchical clustering of Spearman’s correlation of the top 25 HVGs that positively and negatively correlated with *Nr4a1* expression across all cells (SKG and WT). Diagonal gray boxes represent a correlation of 1; dark gray boxes mark distinct gene modules from genes that positively correlated with *Nr4a1* expression. (**B**) UMAP plots show expression levels in all cells of the indicated marker genes positively correlated with *Nr4a1*, as identified in **A**. The scale represents the log-transformed normalized gene counts. (**C**) Volcano plot shows DEGs for SKG and WT cells in the T.4N*_Nr4a1_* cluster that expressed (log-normalized expression >1) *Egr2* or *Tnfrsf9*, with dots colored according to significant overexpression (|log_2_(FC)| >0.5, adj. *P* < 0.05) in *Egr2*-expressing (brown) or *Tnfrsf9* -expressing (teal) cells. (**D**) Enrichment plots from GSEA of GSE17974 data on pathways of time-course in vitro activation of CD4^+^ T cells with anti-CD3 plus anti-CD28 for ranked genes from DEG analysis of cells in the T.4N*_Nr4a1_* cluster that express *Egr2* versus *Tnfrsf9*. (**E**) Heatmap of the average expression of peripheral tolerance defect signature genes from WTNur and SKGNur GFP^hi^ cells expressing *Egr2* or Tnfrsf9 in the T.4N*_Nr4a1_* cluster, normalized by the standard scale for each gene. Teal asterisks next to genes in the heatmap mark significant differential expression between SKGNur GFP^hi^ and WTNur GFP^hi^ cells in the *Tnfrsf9* subcluster.

For a broader examination of genes correlated with *Nr4a1* within our entire dataset, we performed coexpression analysis of highly variable genes (HVGs) in all cells. We identified 3 gene modules of HVGs that positively correlated with *Nr4a1* (Figure 3A). Genes from 2 of these modules, *Egr* family members (immediate early gene transcription factors) and *Tnfrsf9* (4-1BB — the TCR-inducible costimulatory receptor), identified distinct subclusters of cells within the T.N4*_Nr4a1_* cluster (Figure 3B and Supplemental Figure 4A). Cell-cycle stages did not fully account for the division between *Egr* family members and *Tnfrsf9* expression (Supplemental Figure 4B). We found that *Egr2^+^* cells expressed genes induced early after TCR stimulation (including *Egr1*, *Egr2*, *Cd69*, *Ier2*, *Egr3*, *Nfkbid*, *Junb*, *Fos, Myc*, *Cd40lg*), whereas the *Tnfrsf9^+^* cells expressed genes enriched in pathways induced in response to prolonged TCR signaling (e.g., *Pou2f2, Myb, Tnfrsf4, Lag3*) (Figure 3, C and D, Supplemental Figure 4C, and Supplemental Data Files 3 and 6). Moreover, the *Tnfrsf9^+^* cells more robustly induced a previously identified TCR activation gene module compared with *Egr2^+^* cells (36) (Supplemental Figure 4, D and E). These findings suggest that T.N4*_Nr4a1_* cells segregated into subclusters driven by their TCR signaling kinetics (early vs. prolonged stimulation). A subset of tolerogenic genes identified in Figure 2G were also induced at lower magnitudes in SKGNur GFP^hi^ *Tnfrsf9^+^* T cells compared with the corresponding WT subset (Figure 3E). This suggests that SKGNur GFP^hi^ CD4^+^ T cells exhibit a defect in the expression of tolerogenic gene programs in response to prolonged TCR stimulation, rather having a reduced frequency of such cells.

### Cell states and trajectories of T.4N_Nr4a1_ cells have a distinct distribution in the SKGNur GFP^hi^ subset.

**Figure 4:**
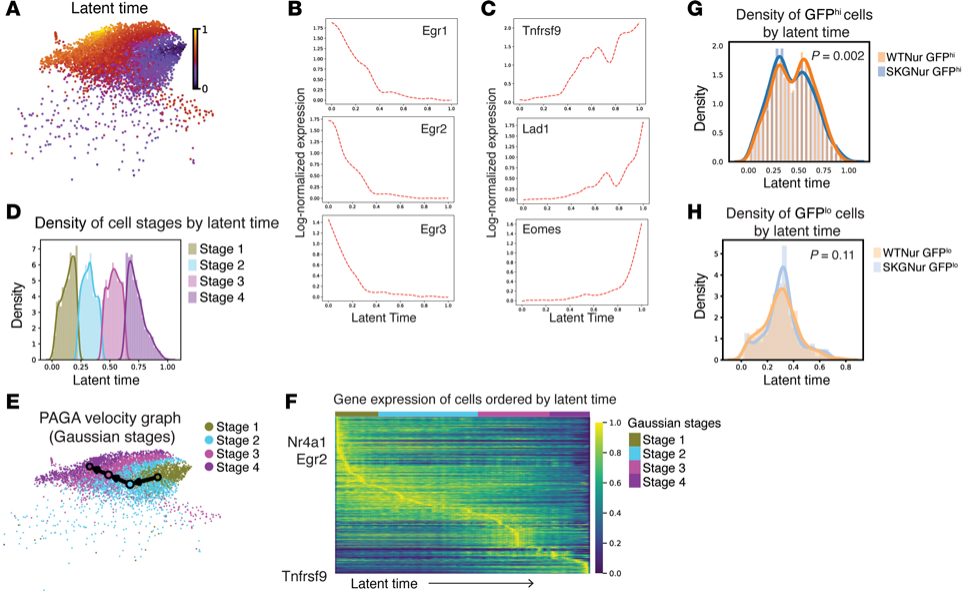
Trajectory analysis of T.4N_Nr4a1_ cells orthogonally uncovers acute versus chronically antigen-activated T cell states with a distinct distribution in the SKGNur GFP^hi^ subset. (**A**) UMAP of cells from the T.4N*_Nr4a1_* cluster colored according to latent time. (**B** and **C**) Smoothed gene expression analysis of cells in the T.4N_Nr4a1_ cluster of selected genes with the highest expression earlier (**B**) or later (**C**) along the latent time axis. (**D**) Probability densities of latent time distribution of cells from the T.4N*_Nr4a1_* cluster assigned to 4 distinct clusters (labeled stages 1–4) by a Gaussian mixture model. (**E**) Predicted transitions from the partition-based graph abstraction (PAGA) algorithm between cells from the stages indicated in **D**. (**F**) Heatmap of single-cell, standard scale–normalized expression of genes ordered top to bottom by peak expression at earlier to later latent times. Chosen genes are the top 300 highest-confidence genes used in the modeling of latent time. Column annotation bar indicates stage assignment of the cell in each column. (**G** and **H**) Probability densities of latent time distribution for GFP^hi^ (**G**) and GFP^lo^ (**H**) cells from WTNur and SKGNur mice, with *P* values determined by Kolmogorov-Smirnov test.

We explored whether the early versus prolonged TCR signaling states in the T.N4*_Nr4a1_* cluster represented endpoints of a trajectory. To this end, we identified a continuum of cell states in the T.N4*_Nr4a1_* cluster ordered by latent time using RNA velocity (49) (Figure 4A). Expression of the *Egr* family peaked in earlier latent-time cells, whereas the expression of *Tnfrsf9* and associated genes peaked in later latent-time cells (Figure 4, B and C).

We used a Gaussian mixture model (50) to deconvolute the distribution of all cells across latent time into 4 cell states labeled stages 1–4 from earlier to later latent time (Figure 4D and Supplemental Figure 4F). The number of underlying distributions, or stages, was determined using the Bayesian information criterion (BIC) and Akaike information criterion (AIC) as clustering validity metrics, and the resulting stage labels had an average silhouette score of 0.554, indicating good-quality clusters (Supplemental Figure 4G). The RNA velocity vector field (Supplemental Figure 4H) and trajectory inference analysis (51) supported a trajectory from stage 1 to stage 4 (Figure 4E). The expression of *Egr2* and *Nr4a1* peaked within cells from stage 1, while the expression of *Tnfrsf9* peaked within cells from stage 4 (Figure 4F and Supplemental Data File 7). The genes overexpressed in stage 1 and stage 4 cells were enriched for early and prolonged TCR stimulation pathways, respectively (Supplemental Figure 4I). Thus, these cell states seemed to be the endpoints of a trajectory of cell states from early to prolonged TCR stimulation.

Cells from the SKGNur GFP^hi^ and WTNur GFP^hi^ groups had significantly different distributions across latent times, with a higher density at the earlier latent time for the SKGNur GFP^hi^ cells, which also had an increased odds of being in stage 1 versus stage 4 compared with WTNur GFP^hi^ cells (OR = 1.25, *P* = 0.02). This difference was not observed between the GFP^lo^ subgroups (Figure 4, G and H).

We hypothesized that this imbalance may reflect either slower progression of SKG cells through the stages or higher input, due to higher proliferation, into stage 1 in the SKG pool. There was no significant difference in cell-cycle distribution between SKGNur GFP^hi^ and WTNur GFP^hi^ cells in stage 1, suggesting that the SKGNur GFP^hi^ cells in stage 1 did not show greater proliferation. While this result favors our slower progression hypothesis, the 2 hypotheses are not mutually exclusive. Slower progression of the SKGNur GFP^hi^ cells would suggest that SKG CD4^+^ T cells have a defect in peripheral tolerance induction — a program that is activated as the cells progress through the stages — and could explain the reduced frequency of anergic cells we previously observed in SKG mice (13).

### SKGNur GFP^hi^ CD4^+^_naive_ T cells demonstrate a biased TCR β variable gene repertoire.

**Figure 5:**
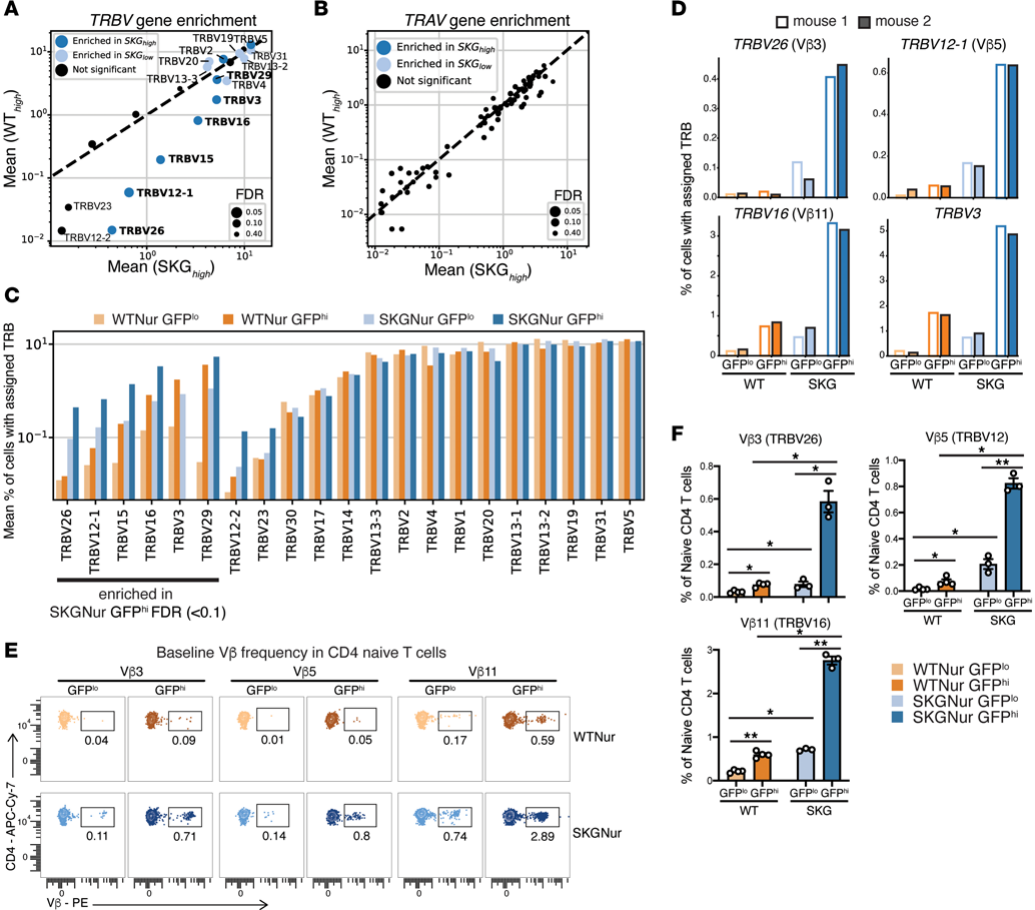
SKG CD4^+^ T cells harbor a biased TCR Vβ gene repertoire. (**A** and **B**) Scatterplot of the mean frequency of cells expressing each *TRBV* (**A**) or *TRAV* (**B**) gene for the SKGNur GFP^hi^ samples versus the WTNur GFP^hi^ samples. Dots for each *TRBV* and *TRAV* genes are sized according to the FDR using a 1-sided, 1-tailed paired *t* test with B-H correction comparing frequencies in SKGNur GFP^hi^ versus SKGNur GFP^lo^ cells. Dots are colored as either significantly enriched (FDR <0.1) in SKGNur GFP^hi^ cells (dark blue), significantly enriched in SKGNur GFP^lo^ cells (light blue), or not significantly enriched in either cell subgroup (black). Dots for significant *TRBV* genes are labeled with the *TRBV* gene name. Labels for *TRBV* genes that were significantly enriched in SKGNur GFP^hi^ cells and were also more highly expressed in SKGNur GFP^hi^ samples versus WTNur GFP^hi^ samples are bolded. (**C**) Bar plot of the mean value of cells expressing each *TRBV* gene as a percentage of all cells in each sample with an assigned *TRBV*. Bars are colored according to subgroup and ordered with the *TRBV* genes enriched in SKGNur GFP^hi^ cells from **A**, followed by the other *TRBV* genes ordered by increasing overall frequency. (**D**) Bar plots of the frequency of cells for each of the 2 replicate mice in each subgroup expressing the indicated *TRBV* genes which were significantly enriched in SKGNur GFP^hi^. (**E** and **F**) Representative FACS plots (**E**) of naive peripheral CD4^+^ T cells with the indicated TCR Vβ protein usage determined by flow cytometry in GFP^lo^ and GFP^hi^ T cells from LNs of WTNur and SKGNur mice prior to arthritis induction and quantification (**F**), where bar graphs depict the mean frequency (± SEM). *n* = 3–4 mice per group. The experiment was repeated at least 3 times. *P < 0.05 and **P < 0.01, for FDR (2-tailed paired Student’s *t* test with B-H correction) or *P* value (exact permutation test).

We previously demonstrated that SKGNur GFP^hi^ cells exhibit heightened self-reactivity and can proliferate in response to unknown endogenous antigens (13). This led us to investigate how their TCR repertoire influences their activation in the periphery. With scTCR-Seq (Figure 2A), we detected paired TCR α (*TRA*) and TCR β (*TRB*) genes in 86% of cells (Supplemental Figure 5A). We did not find oligoclonal expansion in the naive T cells (Supplemental Data File 8). Instead, we found that SKGNur GFP^hi^ T cells had a biased TCR β variable (*TRBV*) gene usage, but not TCR α variable (*TRAV*) usage (Figure 5, A–C, and Supplemental Figure 5B). SKGNur GFP^hi^ CD4^+^_naive_ T cells had significantly higher usage of *TRBV26* (corresponding to TCR variable β 3 [Vβ3] protein), *TRBV12-1* (Vβ5), *TRBV15* (Vβ12), *TRBV16* (Vβ11), *TRBV3*, and *TRBV29* (Vβ7*)* compared with paired SKGNur GFP^lo^ cells (FDR <0.1). Each of these *TRBV* genes also had a higher mean frequency in SKGNur GFP^hi^ cells compared with WTNur GFP^hi^ cells (Figure 5, A, C, and D).

Polyclonal Vβ expansion occurs in the presence of Sags in both humans and mice (52, 53). The *TRBV* genes enriched in SKGNur GFP^hi^ T cells mark Vβs that recognize ERV Sag from MMTV (Supplemental Table 1) (54–56). We confirmed that our SKG colony harbored all 3 endogenous MMTV proviruses (*Mtv-6*, *Mtv-8*, *Mtv-9*) known to be present in BALB/c mice (54, 55, 57, 58) (Supplemental Figure 5C). Exogenous MMTV infection can stimulate cell proliferation and facilitate infection by increasing the number of cell targets, but Sag expression from endogenous *Mtv* leads to clonal T cell deletion in the thymus and resistance to infection owing to the absence of these Sag-reactive, Vβ-expressing T cells (59). However, because of an impairment in SKG TCR signaling, thymic clonal T cell deletion in response to endogenous *Mtv* Sag is incomplete (15), allowing for partial escape of these Sag-reactive T cells into the periphery. In contrast to the *TRBV* genes uniquely enriched in SKGNur GFP^hi^ cells, *TRBV* genes for Vβs that do not recognize *Mtv* Sags in BALB/c (such as *TRBV19*/Vβ6, *TRBV13-2*/Vβ8, *TRBV31*/Vβ14) were not enriched in SKGNur GFP^hi^ T cells (Figure 5C and Supplemental Figure 5D). These results show that negative selection was defective in SKG mice and that encounter with endogenous *Mtv* Sag in the periphery further biased the *TRBV* repertoire in SKGNur GFP^hi^ CD4^+^ T cells.

SKGNur GFP^hi^ cells within the T.N4*_Nr4a1_* cluster also showed enrichment of several Sag-reactive TCRs (*TRBV15* [Vβ12], *TRBV16* [Vβ11], *TRBV29* [Vβ7]) (Supplemental Figure 6, A–D). This enrichment, occurring without TCRα restriction, suggests activation and expansion due to Sag exposure, which contrasts with the typical deletion of Sag-reactive T cells following peripheral *Mtv* Sag encounter (60).

### SKGNur GFP^hi^ CD4^+^ T cells are enriched for Vβs driven by MMTV Sag(s).

To validate our scTCR-Seq results, we assessed TCR Vβ protein levels in SKGNur and WTNur peripheral CD4^+^ T cells before arthritis onset using antibodies against selected Vβs (gating strategy is shown in Supplemental Figure 7A). We found that Vβ protein levels mirrored the transcript abundances from our scTCR-Seq dataset. Vβ3, Vβ5, and Vβ11 (corresponding to *TRBV26*, -*12*, and -*16*, respectively) were significantly enriched in SKGNur GFP^hi^ naive CD4^+^ T cells from lymph nodes (LNs) (Figure 5, E and F) and spleen, whereas non-MMTV Sag targets like Vβ6, Vβ8, and Vβ14 (corresponding to *TRBV19*, -*13*, and -*31*, respectively) were not enriched in SKGNur GFP^hi^ cells (Supplemental Figure 7, B and C).

**Figure 6:**
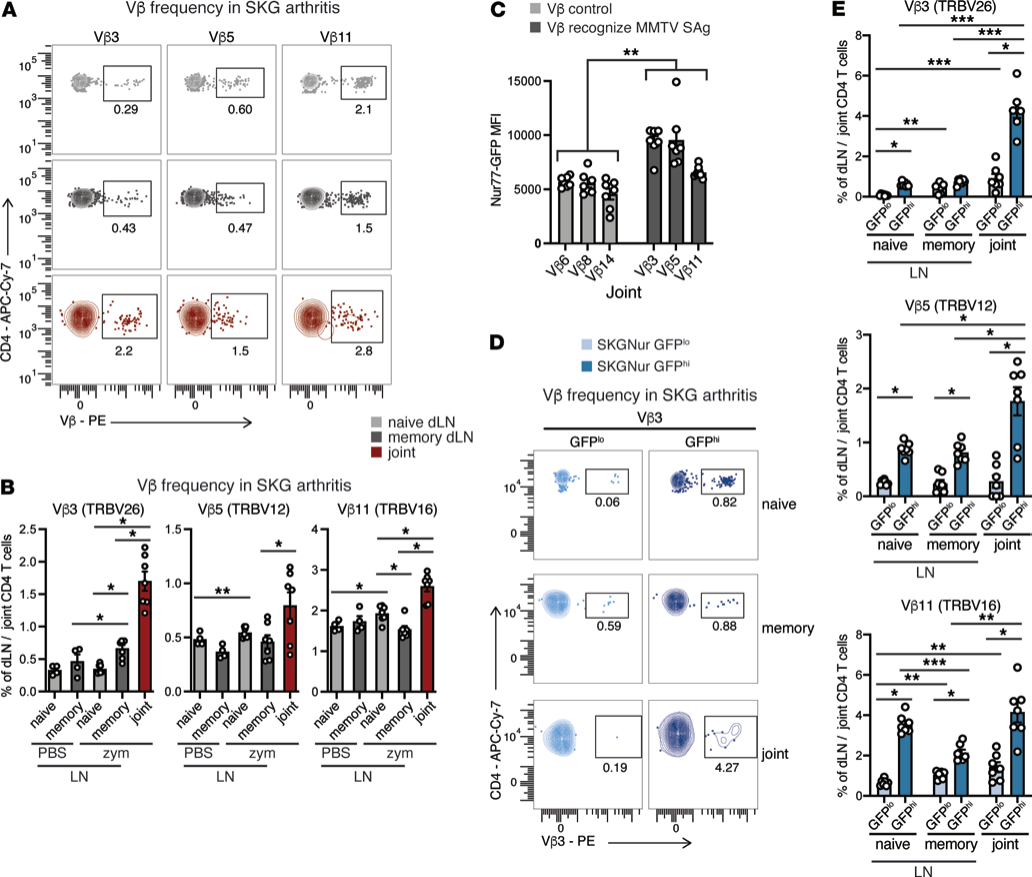
Arthritogenic CD4^+^ T cells are enriched for TCR Vβs that are likely driven by endogenous Sags. (**A** and **B**) Representative FACS plots (**A**) of peripheral naive or memory CD4^+^ T cells or joint CD4^+^ T cells, with the indicated TCR Vβ protein usage determined by flow cytometry in CD4^+^ T cells from dLNs or joints of SKGNur mice 2.5 weeks after arthritis induction with zymosan or mice treated with PBS vehicle (as seen in Supplemental Figure 8B) and quantification (**B**), where bar graphs depict the mean frequency (± SEM). (**C**) Bar graphs showing the GFP MFI (± SEM) of CD4^+^ T cells bearing the indicated Vβs from arthritic joints of SKG mice. *n* = 7 mice pooled from 2 experiments (also reported in Supplemental Figure 8D). (**D** and **E**) Representative FACS plots of (**D**) peripheral naive or memory CD4^+^ T cells or joint CD4^+^ T cells with the indicated TCR Vβ protein usage determined by flow cytometry in GFP^lo^ (light blue) and GFP^hi^ (dark blue) T cells from LNs or joints of SKGNur mice 2.5 weeks after arthritis induction with zymosan and quantification (**E**), in which bar graphs depict the mean frequency (± SEM). *n* = 7 mice per group pooled from 2 experiments. *P < 0.05, **P < 0.01, and ***P < 0.001, for FDR by 2-tailed paired *t* test with B-H correction; *P* value by exact permutation test (**B** and **E**); or FDR by linear mixed-effects model with B-H correction (**C**).

Vβ enrichment in the SKGNur GFP^hi^ T cell subset may be driven by Sag encounter in the periphery and even in joints. Indeed, we detected BALB/c-specific *Mtv* proviruses in SKG mouse joints (Supplemental Figure 8A). Therefore, it is possible that intra-articular *Mtv* Sag expression could engage and enrich for SKG T cells uniquely expressing these Sag-reactive Vβs (Vβ3, Vβ5, and Vβ11) during arthritis. To investigate this possibility, we used zymosan to induce moderate-to-severe inflammatory arthritis in SKG mice (Supplemental Figure 8B) and examined Vβ usage in CD4^+^ T cells harvested from regional joint draining LN (dLNs) and arthritic joints compared with CD4^+^ T cells from the joint dLNs in nonarthritic mice (treated with PBS). We found an increased frequency of MMTV Sag-reactive Vβ3, Vβ5, and Vβ11 in the arthritic joints compared with the periphery (Figure 6, A and B), but not of the non-Sag-reactive Vβs (control Vβs) (Supplemental Figure 8C). Zymosan had a limited effect on Sag-reactive and non-Sag-reactive Vβ frequencies in peripheral naive and memory CD4^+^ T cells (Figure 6B and Supplemental Figure 8C). The Sag-reactive CD4^+^ T cells had a significantly higher Nur77-EGFP MFI compared with CD4^+^ T cells with control Vβs in SKG arthritic joints (Figure 6C) as well as in peripheral naive CD4^+^ T cells (Supplemental Figure 8D). Elevated Nur77 levels in Sag-reactive T cells remained unaffected by zymosan exposure (Supplemental Figure 8D), consistent with our previous findings in human CD4^+^ T cells (22). Furthermore, we observed a significantly higher frequency of Sag-reactive Vβ3, Vβ5, and Vβ11 in SKGNur GFP^hi^ T cells infiltrating the arthritic joints compared with intra-articular GFP^lo^ cells or with GFP^hi^ cells from dLNs (Figure 6, D and E, and Supplemental Figure 8E). This further enrichment suggests that Sag-reactive CD4^+^ T cells expanded after encounter with intra-articular antigen in SKG inflamed joints. We did not observed this enrichment in the joint in SKGNur GFP^hi^ T cells expressing control Vβs (Supplemental Figure 8, F and G).

### Sag-reactive SKG CD4^+^ T cells exhibit impaired tolerance induction, dampened signaling, and a propensity to differentiate into Th17 cells.

**Figure 7:**
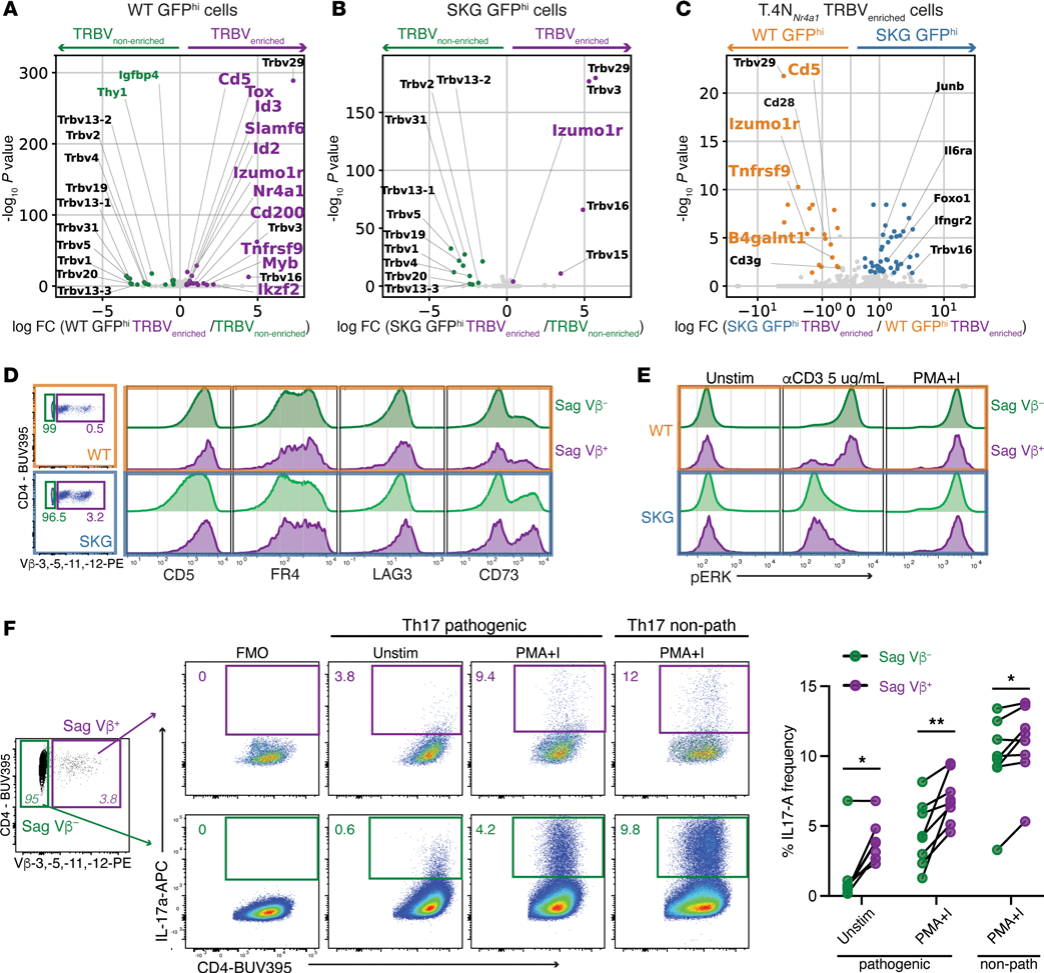
Sag-reactive SKG CD4^+^ T cells show impaired tolerance, defective signaling, and a Th17 differentiation bias. (**A** and **B**) Volcano plots of DEGs within WT (**A**) or SKG (**B**) GFP^hi^ subsets for comparison of Sag-reactive TRBV_enriched_ cells with non-Sag-reactive TRBV_nonenriched_ cells with clored dots indicating significant overexpression (|log_2_(FC)| >0.4, adj. *P* < 0.05) in TRBV_enriched_ (purple) or TRBV_nonenriched_ (green). (**C**) Volcano plots of DEGs within the TRBV_enriched_ cells in the T.4N_Nr4a1_ cluster for comparison to WT or SKG GFP^hi^ mice with dots colored by significant overexpression (|log_2_(FC)| >0.4, adj. *P* <0.05) in WT GFP^hi^ (orange) or SKG GFP^hi^ (blue) cells. (**D**) Left panel: Representative FACS plots of the gating strategy for peripheral CD4^+^CD25^–^ naive Sag-reactive (Vβ3^+^Vβ5^+^Vβ1^+^Vβ12^+^) and Sag^–^ (Vβ3^–^Vβ5^–^Vβ11^–^Vβ12^–^) T cells from LNs of WT or SKG mice. Right panel: Histograms showing surface marker expression in unstimulated cell subsets (quantified in Supplemental Figure 9B). Data represent 6 mice per genotype from 4 independent experiments. (**E**) Histograms display phosphorylated ERK (pERK) levels in Sag-reactive and non-Sag-reactive CD4^+^CD25^−^ T cells gated on naive markers (CD62L^hi^CD44^lo^). Cells were from WT and SKG mice after TCR crosslinking for 2 minutes with anti-CD3ε (αCD3) or stimulation with PMA plus ionomycin (PMA+I). Data represent at least 4 mice per group from 3 independent experiments. (**F**) Left panel: FACS plots show IL-17^+^ cell frequencies in Sag-reactive and non-Sag-reactive CD4^+^ T cells after restimulation with PMA plus ionomycin or vehicle control. Naive CD4^+^CD25^–^ T cells from SKG mouse LNs were cultured for 4 days under pathogenic or nonpathogenic (non-path) Th17 conditions. Right panel: Quantification of mean IL-17^+^ cell frequencies in Sag-reactive (Sag Vβ^+^) and Sag-negative (Sag Vβ^–^) CD4^+^ T cell subsets. Results represent the mean ± SEM. *n* = 8 independent biological replicates per condition (each dot represents data pooled from 2 mice). The experiment was repeated 3 times. *P < 0.05 and **P < 0.01, by 2-tailed paired *t* test. Unstim, unstimulated.

We next compared the transcriptional profiles of *Mtv* Sag-reactive TRBVs with non-Sag-reactive TRBVs, focusing on the effect of peripheral Sag encounter. WT GFP^hi^ cells with Sag-reactive TRBVs (WT GFP^hi^ TRBV_enriched_) compared with WT GFP^hi^ cells with non-Sag-reactive TRBVs (WT GFP^hi^ TRBV_nonenriched_) had significantly higher expression levels of genes (*Tnfrsf9*, *Cd200*, *Ikzf2*, *Myb*) (Supplemental Data Set 9) that are part of the *Tnfrsf9* subcluster/chronic activation signature described in Figure 3. In contrast, we found that, compared with WT, SKG GFP^hi^ cells with *Mtv* Sag-reactive TRBVs (TRBV_enriched_) compared with SKG GFP^hi^ cells with non-Sag-reactive TRBVs (SKG GFP^hi^ TRBV_nonenriched_) had either no significant difference in expression or diminished induction of TCR response genes associated with tolerance and negative regulation of TCR signaling (*Izumo1r*, *Cd5*, *Tox*, *Cd200*, *Tnfrsf9*) (Figure 7, A and B, and Supplemental Data File 9). Additionally, we found that WT GFP^hi^ TRBV_enriched_ cells from cluster T.4N*_Nr4a1_* were significantly more likely than the WT GFP^hi^ TRBV_nonenriched_ cells to be part of the *Tnfrsf9* subcluster rather than the *Egr2* subcluster (OR = 1.5, *P* = 0.04), while there was no significant difference in distribution for those cells from SKG GFP^hi^ mice (Supplemental Figure 9A). We then compared the transcriptional programs of WT and SKG GFP^hi^ TRBV_enriched_ cells within the T.4N*_Nr4a1_* cluster, which are indicative of recent antigen encounter. WT GFP^hi^ TRBV_enriched_ cells expressed higher gene levels of negative regulators of TCR signaling such as *Izumo1r*, *Tnfrsf9*, and *Cd5* compared with their SKG counterparts, which instead showed higher expression of *Foxo1* and *Il6r* (Figure 7C and Supplemental Data File 9). *Mtv* Sags typically induce anergy in Sag-reactive T cells that escape thymic and peripheral deletion (61, 62). In WT mice, most of these cells were eliminated, and those that persisted effectively induced a transcriptional tolerance program (Figure 7, A and C). However, in SKG mice, these cells not only expanded and persisted (Figure 6) but also failed to activate a robust tolerance program despite ongoing peripheral Sag encounter (Figure 7, B and C, and Supplemental Data File 9).

We next examined the surface expression of CD5, FR4, LAG3, CD73, and additional negative regulators of TCR signaling induced after antigen encounter. To capture a larger number of cells, we analyzed these markers in the total Sag-reactive population of naive CD4^+^ cells from SKGNur and WTNur mice. Thus, we were no longer focusing on the GFP^hi^ subsets from our transcriptional analysis, yet we still found that Sag-reactive (Sag Vβ^+^) naive CD4^+^ T cells in both WT and SKG mice expressed higher levels of several inhibitory surface markers compared with non-Sag-reactive (Sag Vβ^−^) T cells (Figure 7D and Supplemental Figure 9B). This likely indicates a compensatory tolerance mechanism to constrain self-reactive T cells, similar to published data on Nur77-GFP^hi^ T cells (13, 63). In results that supported our transcriptional analysis, we detected significantly lower surface expression of CD5 and a trend toward lower levels of FR4/*Izumo1r* expression in SKG Sag-reactive T cells compared with WT cells (Figure 7D and Supplemental Figure 9B). Moreover, WT Sag-reactive T cells exhibited a reduced response to TCR stimulation compared with WT non-Sag-reactive T cells that was probably due to chronic antigen exposure (Figure 7E). This dampening effect was less pronounced in SKG T cells, possibly due not only to their impaired proximal signaling defect, but also to their impaired tolerance mechanisms, which resulted in minimal differences in signaling between Sag-reactive and non-Sag-reactive SKG T cells (Figure 7E). We then assessed the capacity of Sag-reactive T cells from SKG mice to differentiate into IL-17–producing CD4^+^ T cells under both pathogenic and nonpathogenic Th17 conditions. Th17 cells can be categorized as either pathogenic, associated with inflammation and autoimmune disease, or nonpathogenic, associated with tissue homeostasis based on the cytokines present in their microenvironment (64, 65). Pathogenic Th17 cells are driven by signals from TGF-β3 and IL-6 or a combination of IL-6, IL-23, and IL-1β, whereas nonpathogenic Th17 cells are induced by TGF-β1 and IL-6 (65). We observed a higher frequency of IL-17^+^ CD4^+^ T cells among SKG Sag-reactive (Sag Vβ^+^) cells compared with non-Sag-reactive (Sag Vβ^–^) cells in both pathogenic and nonpathogenic conditions (Figure 7F).

### Antiretroviral therapy ameliorates SKG arthritis.

**Figure 8:**
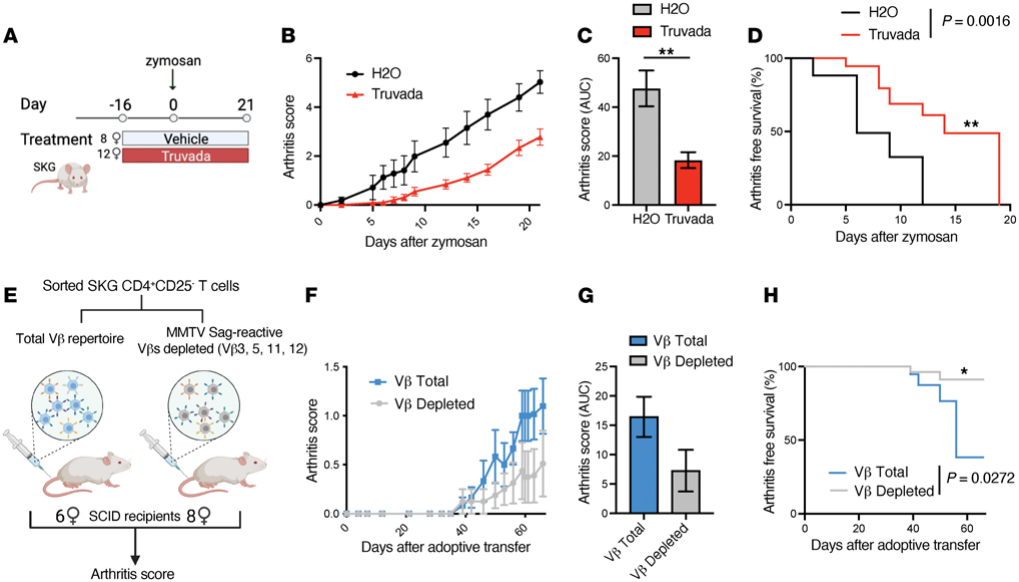
Arthritis pathogenicity partially localizes to Sag-reactive SKG T cells. (**A**) Experimental set-up: SKG mice were treated with Truvada (*n* = 12) or vehicle control (*n* = 8) on day –16 prior to arthritis induction, with i.p. administration of zymosan on day 0. (**B** and **C**) Arthritis score for SKG mice after zymosan injection (**B**), with results plotted as the AUC (**C**). (**D**) Arthritis-free survival plotted as a Kaplan-Meier curve for the results from **A** and **B**, which are representative of 2 independent experiments. (**E**) Sorted SKG CD4^+^CD25^–^ T cells of the indicated Vβ T cell populations were adoptively transferred into SCID mice that were monitored for arthritis development. (**F**–**H**) Arthritis score for SCID mice after adoptive transfer (**F**) and plotted as the AUC (*P* = 0.08) (**G**) and probability of arthritis-free survival (**H**). *n* = 6–8 mice per group. Results are representative of 2 independent experiments**.** *P < 0.05 and **P < 0.01, by 2-tailed Welch’s *t* test (**C** and **G**) or log-rank Mantel-Cox test (**D** and **H**).

We tested whether inhibition of retroviral elements could curtail SKG arthritis and impede Sag-reactive T cell activation and expansion. Mice were treated with Truvada, a combination of the antiretroviral reverse transcriptase inhibitors emtricitabine (Sigma-Aldrich) and tenofovir (Acros Organics), or with vehicle control prior to and during arthritis development (Figure 8A). MMTV reverse transcriptase is sensitive to these compounds, which prematurely terminate nascent cDNA synthesis during reverse transcription and have been reported to decrease viral protein expression (66, 67) and improve inflammatory colitis and autoimmune biliary disease in mouse models (66, 68). We found that Truvada significantly reduced arthritis severity and delayed disease onset in SKG mice (Figure 8, B–D). These data support the idea that ERVs may contribute to arthritis activity in SKG mice. We cannot exclude the possibility that antiretrovirals were inhibiting recombined live MMTV as a result of “endogenous resurrection” — a phenomenon described in immune-compromised mice (69). However, the absence of mammary tumors in aged SKG mice from our colony (Supplemental Figure 9, C and D) suggests that this is unlikely (70).

### Sag-reactive SKG CD4 ^+^T cells are arthritogenic.

We tested whether Sag-reactive CD4^+^ T cells, which comprise approximately 4% of the SKG CD4^+^ T cell population, initiate or augment disease. We performed adoptive transfer of SKG CD4^+^CD25^–^ T cells (Vβ total group) or SKG CD4^+^ T cells depleted of most MMTV Sag-reactive, Vβ-specific cells using available Vβ antibodies (Vβ-depleted) into SCID recipient mice (sorting strategy is shown in Supplemental Figure 9E). Mice that received SKG CD4^+^ T cells depleted of most Sag-reactive Vβs exhibited a significant delay in arthritis onset and a trend toward less severe disease (Figure 8, E–H, and Supplemental Figure 9, F and G). Furthermore, we observed a greater than 5-fold expansion of Sag-reactive Vβs in mice that received Vβ total cells (Supplemental Figure 9H). Together, these results suggest that a portion of the arthritis pathogenicity was contained within the Sag-reactive T cells.

## Discussion

This study explores the gene expression and TCR repertoire in arthritogenic SKGNur GFP^hi^ CD4^+^_naive_ T cells before arthritis onset. Our sequencing analyses revealed that, after antigen exposure and before arthritis onset, these cells showed heightened expression of T cell activation genes (*Nr4a1*, *Egr1-3*, *Irf4*) despite impaired proximal TCR signaling capacity and diminished expression of key tolerogenic markers compared with WT cells.

Our findings provide evidence for a peripheral tolerance breach in arthritogenic SKG CD4^+^ T cells, independent of their central tolerance failure. Normally, TCR activation induces negative regulators that maintain peripheral tolerance and prevent immunopathology (71–74). However, in arthritogenic SKGNur GFP^hi^ CD4^+^ T cells, chronic exposure to endogenous antigens led to incomplete induction of tolerance genes following TCR signaling, unlike the more complete induction of negative regulators observed in WT cells. This compromised signaling in SKG mice failed to fully establish a protective anergy state upon full antigen encounter, as shown in Figures 2 and 7. Additionally, RNA velocity analysis added a temporal perspective and revealed that these arthritogenic SKG cells were predominantly found in early TCR signaling states (stage 1) and failed to advance to stages associated with extended and more effective TCR signaling (stage 4).

Our study reveals that arthritogenic SKG T cells had an enrichment of Sag-reactive T cells. These cells, which escape negative selection in the thymus and evade peripheral deletion by endogenous *Mtv* Sag encounter (60), showed a significant TCR Vβ bias in the peripheral naive SKG repertoire. This bias suggests substantial peripheral Sag engagement. Through tracking of these Sag-reactive T cells by their Vβs, we observed that these cells in WT mice had increased expression of tolerance genes and inhibitory receptors, indicating substantial peripheral Sag engagement. Notably, WT Sag-reactive T cells had a poorer response than non-Sag-reactive T cells to ex vivo restimulation. In contrast, SKG Sag-reactive T cells had reduced expression of inhibitory genes and a subset of inhibitory receptors (on the protein level) and were more prone to differentiate into IL-17–producing cells.

The peripheral Vβ bias extends to SKG arthritic joints, indicating expansion due to intra-articular *Mtv* Sag encounter. While alternative joint-specific endogenous antigen responses cannot be ruled out, our data strongly suggest that the expansion of Sag-reactive T cells was closely associated with intra-articular *Mtv* Sag interactions, as evidenced by significantly higher levels of Nur77-EGFP in Sag-reactive T cells infiltrating the joint (*P* < 0.01). The potential role of *Mtv* Sag in arthritis development is underscored by our findings that ERV reverse transcriptase inhibitors (such as Truvada) significantly mitigated SKG arthritis, probably by disrupting the effect of ERVs on innate and adaptive immune responses (67, 75, 76) and possibly influencing Sag-reactive T cell activation. Furthermore, depletion of Sag-reactive T cells curtailed SKG arthritis, highlighting the pivotal role of endogenous Sag in disease progression. Our results also emphasize the crucial role of chronic antigen encounter in breaking peripheral tolerance in T cells with impaired signaling, leading to their activation and differentiation into pathogenic effector cells.

**Figure 9:**
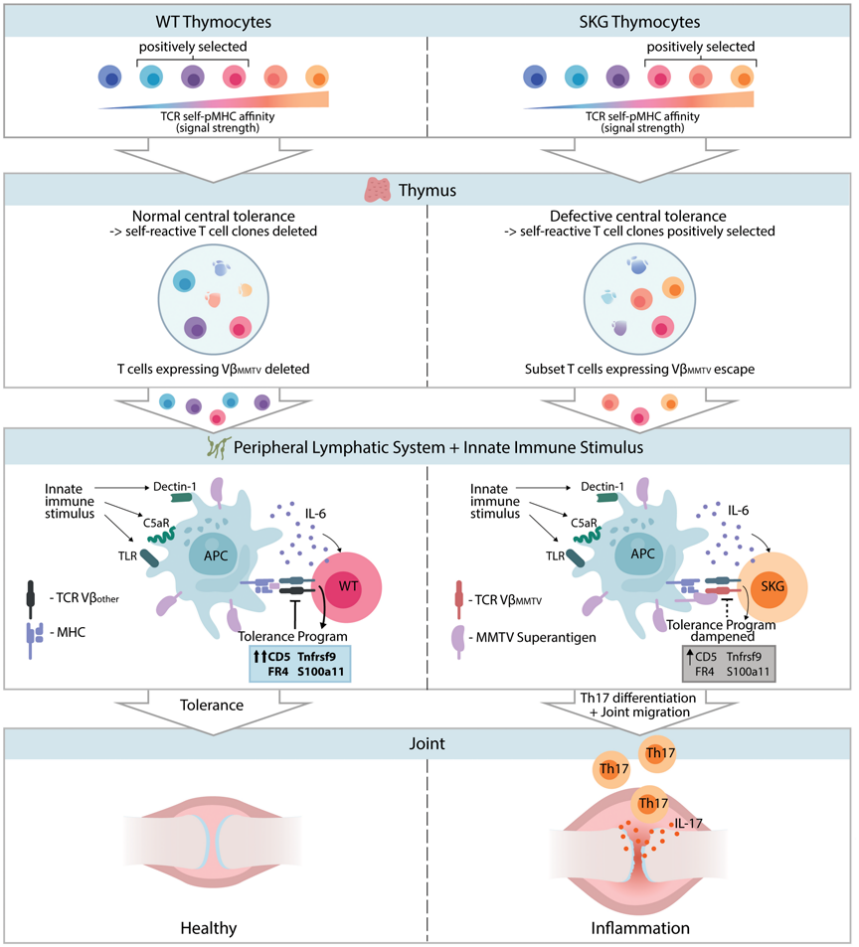
Sag-reactive SKG T cells evade central and peripheral tolerance and contribute to SKG arthritis. Impaired TCR signaling in SKG mice leads to a more self-reactive repertoire and escape of autoreactive, along with MMTV Sag-reactive, CD4^+^ T cells into the periphery. Chronic encounter with peripheral antigens and innate immune stimuli activates these T cells (identified as GFP^hi^ cells) via their TCR. As a result of impaired TCR signal transduction, SKG mice show reduced induction of TCR negative regulators and fail to have a fully established protective anergy state upon antigen encounter. Consequently, in the setting of certain environmental cues (e.g., IL-6 signaling), SKG T cells encountering endogenous antigens differentiate into pathogenic IL-17–producing effector T cells that cause erosive arthritis.

Our proposed model (Figure 9) synthesizes our current and previous findings (12, 13, 15, 77) to explain the complex interplay between compromised TCR signaling, inefficient negative selection, chronic antigen encounter, and altered peripheral tolerance in autoimmunity. This interplay leads to a breakdown in tolerance, characterized by reduced induction of negative immune regulators and fewer anergic cells (13). The compromised TCR signaling threshold in Tregs, coupled with changes in their repertoire, exacerbates this loss of tolerance (14). Molecules like IL-6 may act as costimulatory signals, enhancing T cell survival and lowering their threshold for activation and differentiation. The biased self-reactive TCR Vβ repertoire in SKGNur GFP^hi^ CD4^+^ T cells, along with their activated state, primes these cells to respond to innate immune stimuli, potentially initiating or propagating disease (Figure 9). Our findings show that some of the pathogenicity was contained within Sag-reactive T cells and that antiretroviral therapy significantly reduced SKG arthritis development (*P* < 0.01). Future studies will involve a direct investigation of the role of Sag-reactive Vβs, *Mtv* Sags, and ERVs in SKG arthritis and their relevance to human autoimmune disorders (76, 78–81), including RA (78–80, 82, 83) and other autoimmune arthritides (84–88), in which specific Vβ-expressing T cells expand and persist in the synovial microenvironment.

## Methods

### Sex as a biological variable.

Female mice were used in the sequencing studies and arthritis experiments because of their higher disease penetrance and severity (12). Both sexes were used in the signaling studies, and sex was not controlled as a biological variable in those experiments.

### Mice.

BALB/c and C57BL/6J mice were purchased from The Jackson Laboratory, and BALB/cNur77-EGFP and SKGNur77-EGFP mice were bred in our facility (UCSF) as previously described (13). All mice were housed and bred in specific pathogen–free conditions in the Animal Barrier Facility at UCSF according to the University Animal Care Committee and NIH guidelines.

### Murine synovial tissue preparation.

See Supplemental Methods.

### Antibodies and reagents.

See Supplemental Methods.

### Surface and intracellular staining.

See Supplemental Methods.

### Nuclei isolation and NFAT nuclear staining.

See Supplemental Methods. The nuclei isolation protocol was adapted from the supplemental methods in ref. 89 and methods from ref. 90.

### In vivo treatments.

Power analyses were performed on the basis of preliminary data to calculate the number of mice needed in each group to reach a power of 0.8 and detect a 50% difference between groups with a SD of 30% and a type I error of 0.05. Adoptive transfer experiments were performed as described previously (12, 13). Negatively selected CD4^+^ T cells from 8- to 12-week-old female SKG mice were sorted on CD4^+^CD25^–^ markers with or without depletion of the following Sag-reactive Vβs: Vβ3, Vβ5, Vβ11, Vβ12 (using Vβ antibodies conjugated with PE), and 4.5 × 10^5^ cells were adoptively transferred by tail vein injection into 8-week-old female SCID recipients. To reduce the potential for a cage effect, female mice were randomized prior to the start of the study to different cages and groups, controlling for litter, cage, and age. Bedding was also mixed between all cages to reduce the effect of potential baseline differences in the microbiome 1 week prior to study’s start date. Mice were housed 2–3 per cage to minimize the cage effect after study induction. The scorer was blinded to the treatment condition in the adoptive transfer studies.

### Th17 differentiation.

See Supplemental Methods.

### Flow cytometry and cell sorting.

Cells were stained with antibodies of the indicated specificities and analyzed on BD LSR Fortessa and BD LSR Fortessa DUAL (cytometer 2, Supplemental Figure 8B) flow cytometers (BD Biosciences). Flow cytometry plots and analyses were performed using FlowJo versions 10.8.0–10.10 (Tree Star). Cells were sorted to greater than 95% purity using a MoFlo XDP (Beckman Coulter).

### PCR and reverse transcription PCR.

See Supplemental Methods.

### Bulk RNA-Seq.

Negatively selected CD4^+^ T cells from the lymph node were sorted for CD62L^hi^CD44^lo^CD25^–^ and the 10% highest (GFP^hi^) or lowest (GFP^lo^) expressing T cells. Cells were washed, pelleted, and immediately flash-frozen using dry ice in ethyl alcohol. Samples were processed for bulk RNA-Seq by Q2 solutions using the TruSeq Stranded mRNA kit (Illumina: RS-122-2103) for library preparation. The resulting libraries were pooled into 3 batches and sequenced on an Illumina HiSeq 2500 sequencer over 3 lanes.

### Alignment and initial processing of bulk RNA-Seq data.

The raw FASTQ files were clipped and filtered using FASTQ-mcf version1.04.636 to remove low-quality reads and bases, homopolymers, and adapter sequences. The filtered reads were aligned using STAR version 2.4 (91) with the default set to the mm10 transcriptome, and the resulting BAM files were converted to count matrices for each sample with RNA-Seq by Expectation-Maximization (RSEM) version 1.2.14. Genes with fewer than 10 counts across all the samples were filtered out. Raw counts were normalized and transformed by the variance-stabilizing transformation (VST) function from DESeq2 version1.22.2 (92).

### PCA analysis.

The VST-normalized features were used for PCA with the function plotPCA from DESeq2.

### Bulk RNA-Seq differential expression.

Differential gene expression analysis of the bulk RNA-Seq samples was performed with the raw counts from the filtered gene list for the indicated samples as the inputs. The analysis was run using a negative binomial model with multiple testing correction with Benjamini-Hochberg (B-H) implemented via the DESeq function, which includes an internal normalization from DESeq2. For differential gene expression between samples within the same genotype, mouse identity was included as a covariate.

### Functional enrichment analysis.

See Supplemental Methods.

### Gene set enrichment analysis.

See Supplemental Methods.

### scRNA-Seq and TCR-Seq.

Negatively selected CD4^+^ T cells from lymph nodes and spleens were sorted for CD62L^hi^CD44^lo^CD25^–^ and the 10% of T cells with the highest (GFP^hi^) or lowest (GFP^lo^) expression of GFP. Droplet-based, paired scRNA- and TCR-Seq was performed using the 10x single-cell 5′+V(D)J version1 kit per the manufacturer’s instructions. The resulting cDNA libraries were sequenced on 4 lanes of an Illumina Novaseq 6000 sequencer to yield gene expression (GEX) and TCR FASTQs.

### Alignment and initial processing of sc-Seq data.

The raw FASTQ files were aligned using CellRanger versions 3.0.1 and 3.0.2 software, with the default set to the mm10 transcriptome with the addition of the sequence for the EGFP transcript and the vdj GRCm38 version 3.1.0 reference for the GEX and TCR FASTQs, respectively.

### EGFP transcript sequence.

See Supplemental Methods.

### Cell type classification and clustering.

See Supplemental Methods.

### Single-cell differential expression analysis.

Single-cell differential expression was performed using the Wilcoxon rank-sum method and multiple testing correction with a B-H test implemented with the rank_genes_groups function from ScanPy on the log-normalized gene counts. Additionally, the adjusted *P* values that were equal to 0 were updated to the minimum representable positive normalized float (2.2250738585072014e-308).

### Cell-cycle phase assignment and module scoring.

See Supplemental Methods.

### RNA velocity analysis.

See Supplemental Methods.

### TCR analysis.

Cells with 2 or fewer TRA chains and 1 or fewer TRB chains were used in the TCR clonotype analyses (52). Cells with 2 TRA chains were removed for the TRBV and TRAV analyses, since the highest frequency for any dual TRA was 0.09% in any 1 sample (~1 cell). This removed 10,598 cells, or 13.6% of all cells, which is consistent with the expected dual TRA frequency. TRBV and TRAV genes that were not present in at least 2 mice from the same subgroup (SKGNur GFP^hi^, WTNur GFP^hi^, SKGNur GFP^lo^, and WTNur GFP^lo^) were removed from the downstream TRBV and TRAV analyses.

### Statistics.

Flow cytometric data were analyzed by comparison of means using paired or unpaired, 2-tailed Student’s *t* tests with GraphPad Prism version 9.2.0 or 9.3.1 for Mac (GraphPad Software). A 2-tailed Welch’s *t* test was used to calculate differences in arthritis scores, and a log-rank Mantel-Cox test used to calculate differences in Kaplan-Meier survival curves.

Significant differences in the TRBV frequencies between subgroups were determined by exact permutation test for unpaired and paired samples (for *n* >5 paired samples) (93) or 1-tailed paired Student’s *t* test with B-H correction (for *n* ≤5 paired samples) using SciPy version 1.4.1 and statsmodels version 0.11.1.

The latent time distributions from different subgroups were compared using the Kolmogorov-Smirnov test. The cell-cycle distributions between subgroups within stage 1 were compared using Pearson’s χ^2^ test.

Significant differences in GFP MFI for cells assigned TRBVs in the enriched or not-enriched groups were determined with a linear mixed-effects model EGFP MFI ~ TRBV group (enriched or not-enriched) plus mouse identity (for paired data), with a random intercept for each TRBV protein followed by B-H correction. Significant differences in MFI for surface protein markers between groups for Sag-reactive and Sag non-reactive cells from WT GFP^hi^ and SKG GFP^hi^ subsets were determined by linear mixed-effects model MFI ~ subgroup for unpaired samples (e.g., WT Sag-reactive vs. SKG Sag-reactive) and MFI ~ Sag type plus mouse for paired samples (e.g., WT Sag-reactive vs. WT Sag non-reactive), with a random intercept for the flow cytometer machine, and *P* values were adjusted using B-H correction for multiple testing. Samples were collected on different cytometers because of a cytometer 1 malfunction. Data in all figures represent the mean ± SEM unless otherwise indicated. Differences were considered significant at a *P* value of less than 0.05.

Odds ratios were calculated using a conditional maximum likelihood (CML) estimator with SciPy version1.6.1.

### Study approval.

All animal experiments were approved by the UCSF’s IACUC (IRB AN192722).

### Data and materials availability.

Bulk RNA-Seq data and scRNA-Seq and scTCR-Seq data discussed in this publication have been deposited in NCBI’s Gene Expression Omnibus (GEO) database (GEO GSE185577; https://www.ncbi.nlm.nih.gov/geo/query/acc.cgi?acc=GSE185577). All other data are available in the main text or as supplemental data. Values for all data points in the graphs are reported in the Supporting Data Values file. Code for analysis is available at: https://github.com/yelabucsf/SKG_rheum (commit ID: 6943b1c).

## Author contributions

JFA, AW, CJY, and JD were responsible for study conceptualization. JFA, CJY, AW, EEM, SY, and YN designed the study methodology. JFA, EEM, SY, CL, NP, YN, RN, and VR conducted experiments. JFA, EEM, and CJY conducted formal analyses. JFA, EEM, SY, CL, NP, YN, and RN were responsible for visualization. JFA, AW, CJY, and JD acquired funding. JFA, AW, CJY, JD, and EEM administered the project. JFA, AW, CJY, and JD provided resources. JFA, AW, and CJY supervised the study. JFA and EEM wrote the original draft of the manuscript. JFA, EEM, AW, and CJY reviewed and edited the manuscript.

## Supplementary Material

Supplemental data

Supplemental data set 1

Supplemental data set 2

Supplemental data set 3

Supplemental data set 4

Supplemental data set 5

Supplemental data set 6

Supplemental data set 7

Supplemental data set 8

Supplemental data set 9

Unedited blot and gel images

Supporting data values

## Version 1 11/26/2024

In-Press Preview

## Version 2 01/16/2025

Electronic publication
