# Supplementary material for: Endogenous antigens shape the transcriptome and TCR repertoire in an autoimmune arthritis model
Source: J Clin Invest. 2024 Nov 26;135(2):e174647. doi: 10.1172/JCI174647 (PMC11735108; doi:10.1172/JCI174647)
Supplement: Supplemental data [file jci-135-174647-s214.pdf]

Supplementary Materials for

Endogenous antigens shape the transcriptome and TCR repertoire in an autoimmune arthritis model

Elizabeth E. McCarthy, Steven Yu, Noah Perlmutter, Yuka Nakao, Ryota Naito, Charles Lin, Vivienne Riekher, Joe DeRisi, Chun Jimmie Ye, Arthur Weiss, Judith F. Ashouri

Correspondence to: Judith.Ashouri@ucsf.edu; Arthur.Weiss@ucsf.edu;

Jimmie.Ye@ucsf.edu

This file includes:

Supplementary Methods

Supplementary Figures S1 to S9

Supplementary Table S1

Captions for Data files S1 to S9

Other Supplementary Materials for this manuscript include the following:

data_S1_bulk_RNA_seq_diff_exp.xlsx,

data_S2_heatmap_gene_list_with_modules.xlsx,

data_S3_GSEA_reports.xls,

data_S4_scRNAseq_diff_genes_by_cluster.xlsx,

data_S5_fig_2_diff_gene_lists.xlsx,

data_S6_fig_3_diff_exp_lists.xlsx,

data_S7_top_300_heatmap_gene_list.tsv,

data_S8_gini_coefficients.xlsx,

data_S9_diff_exp_TRBV_enriched_TRBV_non_enriched.xlsx

Supplementary Methods

Antibodies and reagents. Ghost Dye Violet 510 (Tonbo: 13-0870-T100) was used for live/dead staining. The following antibodies were used for staining as indicated: CD3e-BUV395 (BD Biosciences: 563565, clone: 145-2C11), CD4-APCeFluor 780 (eBiosciences: 47-0042-82, clone: RM4-5) or BUV395 (BD Biosciences: 563790, clone GK1.5) or PE (Tonbo: 50-0041-U100, clone GK1.5), CD25-PerCPCy5.5 (Tonbo 65-0251-U100, clone: PC61.5), CD44-PE-Cy7 (BioLegend: 103030, clone: IM7) or BV421 (BD Biosciences: 563970, clone: IM7), CD62L-BV711 (BioLegend: 104445 or BD Biosciences: 740660, clone: MEL-14), TCR Vβ3-PE (BD Biosciences: 553209, clone: KJ25, or Invitrogen: 25-7311-82, clone KJ25), TCR Vβ5.1/5.2-PE (BD Biosciences: 562088, clone: MR9-4, or BioLegend: 139504, clone MR9-4), TCR Vβ6-BV421 (BD Biosciences: 744590, clone: RR4-7), TCR Vβ8-BV421 (BD Biosciences: 742376, clone: F23), TCR Vβ11-PE (BD Biosciences: 553198, clone: RR3-15), TCR Vβ12-PE (BioLegend: 139704, clone MR11-1), TCR Vβ14-Biotin (BD Biosciences: 553257, clone: 14-2), Streptavidin-BV421 (BioLegend: 405226), FOXP3-eFluor 660 (eBioscience: 50-5773-82, clone: FJK-16s), anti-mouse NFAT-Alexa Fluor 647 (Cell Signaling Technologies: 14201S, clone D43B1), rabbit anti-Phospho-p44/42 MAPK (Erk1/2)(Thr202/Tyr204) (Cell Signaling Technologies: 4377S, clone 197G2), donkey anti–rabbit secondary Ab conjugated to Alexa Fluor 647 (Jackson ImmunoResearch: 711-605-152), anti-mouse IL-17A Alexa 647 (BD Biosciences: 560184, clone TC11-18H10), anti-Mouse CD16/CD32 (Fc Shield) (TONBO Biosciences: 70-0161-M001, clone 2.4G2).

The following additional antibodies were used for staining as indicated: anti-mouse CD3 Alexa 780 (Invitrogen, clone 17A2) or PerCP-Cy5.5 (BD Biosciences, clone 145-2C11), anti-mouse CD4 BUV395 (BD Biosciences, clone GK1.5), anti-mouse CD62L BV711 (BioLegend, clone MEL-14), anti-mouse CD44 BV421 (BD Biosciences, clone IM7), anti-mouse CD25 PerCP-Cy5.5 (TONBO Biosciences, clone PC61.5) or BV650 (BioLegend, clone PC61), anti-mouse CD134 (OX-40) PE (BioLegend, clone OX-86), anti-mouse CD137 (4-1BB) APC (BioLegend, clone 17B5), anti-mouse CD69 APC (BioLegend, clone H1.2F3), anti-mouse CD5 PE (BD Biosciences, clone 53-7.3).

Murine synovial tissue preparation. Synovial tissues from ankle joints were digested with 1 mg/mL Collagenase IV (Worthington: LS004188) and DNase I (Sigma: 4536282001) in RPMI 1640 medium for 2 h at 37 °C on a rotator then quenched with 10% fetal bovine serum in RPMI 1640 medium; digested cells were filtrated through a 70 µm nylon mesh to prepare single cell suspensions.

Surface and intracellular staining. After live/dead staining with Ghost Dye Violet 510 as per manufacturer's instructions, cells were stained for surface markers, washed, and then fixed for 10 min with 4% (vol/vol) fresh paraformaldehyde at room temperature protected from light. Cells were then permeabilized using the Mouse Regulatory T-Cell Staining kit 1 (eBioscience: 00-5521-00) per manufacturer's instruction and then stained with FoxP3 e660.

Nuclei isolation and NFAT nuclear staining. Sorted $CD4^{+}CD25^{-}$ naïve ($CD62L^{hi}CD44^{lo}$) T cells from LN and spleen harvested from WTNur and SKGNur mice were pre-warmed for 15 min at 37°C in complete media (RPMI with 10% FBS, glutamine, HEPES, β-ME, sodium pyruvate, and non-essential amino acids). Cells were then transferred to 96-well plate pre-coated with anti-CD3e at 5 μg/mL (clone 145-2C11, Biolegend 100331) at a concentration of 2.0×10^{5} cells/100 µl per well + 2 μg/mL soluble anti-CD28 (clone 37.51, Biolegend 102112) for the indicated time points at 37°C or stimulated with 1 μM ionomycin for 2 hours or vehicle control. Stimulated cells were spun at 600 x *g* at 4°C, and the cells were immediately resuspended with 200 µl of ice-cold Buffer A containing 320 mM sucrose, 10 mM HEPES, 8 mM $MgCl_2$, 1× Roche EDTA-free complete Protease Inhibitor, and 0.1% (v/v) Triton

X-100 (Sigma-Aldrich). After 15 min on ice, the plate was spun at 2000 × *g* and 4°C for 5 min. This was followed by two 200 µl washes with Buffer B (Buffer A without Triton X-100) with gentle resuspension and centrifugation at 2000 × *g* at 4°C for 5 min. After the final wash, pellets were resuspended with 200 µl Buffer B containing 4% paraformaldehyde (electron microscopy grade; Electron Microscopy Sciences), and nuclei were fixed on ice for 30 min. Nuclei were spun at 2000 x *g* at 4°C for 5 min, and followed a wash and resuspension in 200 ul nuclei FACS Buffer (1× PBS with 2% FBS and 8mM $MgCl_2$), centrifuging at 1000 × *g* and 4°C for 5 min to sufficiently pellet nuclei.
Nuclei staining: Isolated nuclei were washed with 180 µl nuclei Perm Buffer (FACS Buffer with 0.3% Triton-X 100) and spun at 1000 × *g* and 4°C for 5 min and then stained with 40 µl anti-mouse NFAT AlexaFluor 647 (Cell Signaling: 14201S, clone D43B1) diluted at 1:400 in Perm Buffer for 60 min on ice. The 96-well plate was spun at 1000 x *g* at 4°C for 5 min and washed with 180 µl nuclei FACS Buffer and followed by a wash of 180 ul nuclei Perm FACS Buffer for the last wash. Nuclei were resuspended in nuclei FACS Buffer and analyzed (while kept on ice) immediately on a BD Fortessa cytometer.

In vivo mouse treatment. Arthritis: Zymosan A (Sigma-Aldrich) suspended in saline at 10 mg/mL was kept in boiling water for 10 min. Zymosan A solution 2 mg or saline was intraperitoneally injected into 8–12-week-old mice. Antiretroviral therapy: 5–7-week-old SKG mice were administered Truvada combination therapy with emtricitabine (Sigma-Aldrich) and tenofovir disproxil fumarate (Acros organics) in a 1:1 ratio (0.5 mg/mL in diH2O for each drug) or vehicle control. Solution was added to drinking water supply and changed once per week. Mice were also given an intraperitoneal bolus injection x1 of Truvada (~160 mg/kg) or vehicle control in 200 ul PBS at start of treatment. Drinking water dosage with Truvada or diH2O continued throughout the arthritis course. Power analysis was performed based on preliminary data to calculate number of mice needed in each group to reach a power of 0.8 and detect a 50% difference between groups with standard deviation of 30% and type I error = 0.05.

PCR and RT-PCR. BALB/cJ, C57BL/6J, and SKG tail DNA was typed for *Mtv-6, -8, -9,* and *-17*. Standard PCR protocols were used for preparing PCR mixtures. Primer pairs for the detection of MMTV proviruses were previously described (*62*). GAPDH primers used: (5' CATGTTTGTGATGGGTGTGAACCA 3') and (5' GTTGCTGTAGCCGTATTCATTGTC 3'). PCR mixtures for *Mtv-6, -8, and -9* were incubated at 94°C for 5 min, then denatured for 44 cycles at 94°C for 1 min, annealed at 46°C for 1 min, polymerized at 72°C for 1 min, and then incubated at 72°C for 5 min. PCRs for *Mtv-17* were conducted similarly except for an annealing temperature of 50°C. Samples were run on 2% agarose gel.

RT-PCR with joints. Single cells suspensions of synovial tissues from SKG ankle joints were spun down at 1500 RPM at 4C. Cell pellets were flash frozen using dry ice in ethyl alcohol. Frozen cell pellets were used with the Rneasy Mini Kit (Qiagen: 74106) for RNA purification. The qScript cDNA Synthesis Kit (Quantabio: 95047-100) was used for cDNA library synthesis from purified total RNA. RT-PCR was conducted as described previously for PCR.

In vitro Th17 differentiation. Pooled peripheral LN (comprising axillary, brachial, inguinal, popliteal and mesenteric) and spleen from SKG mice were isolated for naïve CD4+ T cells by magnetic sorting using EasySep™ Mouse Streptavidin RapidSpheres™ isolation kit (STEMCELL Technologies, USA) with the addition of anti-CD44 biotin (1:10 dilution). Cells were seeded in 24-well plates at $6.25x10^5$/well (coated with 2μg/mL anti-mouse CD3e (145-2C11, BioLegend, San Diego, CA, USA) and soluble anti-mouse CD28 (37.51, BioLegend, San Diego, CA, USA). Non-pathogenic condition for T_H17 cell differentiation was induced by adding 5ng/mL recombinant human TGF-ß (R&D Systems, MN, USA), 25ng/mL recombinant mouse IL-6 (Peprotech, NJ, USA), 5μg/mL anti-mouse IFNγ (BioXCell, NH, USA), and 5μg/mL anti-mouse IL-4 (BioXCell). For pathogenic condition, cells were differentiated by adding 20ng/mL recombinant mouse IL-23 (R&D Systems), 20ng/mL recombinant mouse IL-1ß (R&D Systems), 25ng/mL recombinant mouse IL-6 (Peprotech), 5μg/mL anti-mouse IFNγ (BioXCell), and 5μg/mL anti-mouse IL-4 (BioXCell) for 4 days. On day 2, cells were replenished with fresh T_H17 cell inducing factors at the same concentration. Day 4, cells were

collected, washed, and restimulated with 100ng/mL PMA (Sigma-Aldrich, MO, USA), 1.4μM ionomycin for 4hr at 37°C in the presence of Golgiplug protein transport inhibitor (BD Biosciences, CA, USA). Cells were then washed and stained with anti-mouse CD4 BUV395 (BD Biosciences) antibody. For intracellular staining, cells were fixed and permeabilized using the Cytofix/Cytoperm™ Fixation/Permeabilization kit (BD Biosciences) followed by intracellular staining with anti-mouse IL-17A Alexa 647 (BD Biosciences), anti-mouse TCR Vß3 PE (Invitrogen), anti-mouse TCR Vß5 PE (BioLegend), anti-Mouse TCR Vß11 PE (BD Biosciences), and anti-mouse TCR Vß12 PE (BioLegend) antibodies. Cells were then analyzed by flow cytometry.

Functional enrichment analysis. The collection of 991 significantly differentially expressed genes (log2FC > 1 and adjusted p value < 0.05) from the four comparisons [SKGNur GFP^{hi} versus SKGNur GFP^{lo} , WTNur GFP^{hi} versus WTNur GFP^{lo}, SKGNur GFP^{hi} versus WTNur GFP^{hi}, SKGNur GFP^{lo} versus WTNur GFP^{lo}] were hierarchically clustered using the Ward linkage (“ward.D2”) with the R package pheatmap v.1.0.12. The resulting dendrogram was used to partition the differentially expressed gene list into six gene modules. The gene lists for each gene module were analyzed using the functional profiling g:GOSt tool from g:Profiler (version e102_eg49_p15_e7ff1c9) with g:SCS multiple testing correction method applying significance threshold of 0.05. Select significantly enriched pathways from the GO:BP or KEGG collections were reported.

Gene set enrichment analysis. For the bulk RNA differential expression, the differential gene list was filtered to remove genes with NA for the adjusted p value or log fold change. For the single-cell RNA differential expression, the differential gene list was filtered to only include genes which were expressed in at least 1% of cells in the $T.4N_{Nr4a1}$ cluster. These filtered gene lists were used to create ranked gene lists with the sign(log fold change) times the -log10(raw p value) as the ranking metric. The ranked list was used as input to look for gene set enrichment in the indicated collection of pathways in the ‘classic’ mode with the GSEAPreranked tool from GSEA v.4.1.0 with the default settings. For pathway collections of human genes, the ‘Mouse_Gene_Symbol_Remapping_Human_Orthologs_MsigDB’ chip file was used to map mouse

genes from the ranked gene list to the human orthologs. Mouse gene symbols that mapped to the same human symbol were collapsed based on the max rank.

eGFP transcript sequence.

ATGGTGAGCAAGGGCGAGGAGCTGTTCACCGGGGTGGTGCCCATCCTGGTCGAGCTGGACGG
CGACGTAAACGGCCACAAGTTCAGCGTGTCCGGCGAGGGCGAGGGCGATGCCACCTACGGCA
AGCTGACCCTGAAGTTCATCTGCACCACCGGCAAGCTGCCCGTGCCCTGGCCCACCCTCGTGA
CCACCCTGACCTACGGCGTGCAGTGCTTCAGCCGCTACCCCGACCACATGAAGCAGCACGACT
TCTTCAAGTCCGCCATGCCCGAAGGCTACGTCCAGGAGCGCACCATCTTCTTCAAGGACGACG
GCAACTACAAGACCCGCGCCGAGGTGAAGTTCGAGGGCGACACCCTGGTGAACCGCATCGAGC
TGAAGGGCATCGACTTCAAGGAGGACGGCAACATCCTGGGGCACAAGCTGGAGTACAACTACA
ACAGCCACAACGTCTATATCATGGCCGACAAGCAGAAGAACGGCATCAAGGTGAACTTCAAGAT
CCGCCACAACATCGAGGACGGCAGCGTGCAGCTCGCCGACCACTACCAGCAGAACACCCCCAT
CGGCGACGGCCCCGTGCTGCTGCCCGACAACCACTACCTGAGCACCCAGTCCGCCCTGAGCAA
AGACCCCAACGAGAAGCGCGATCACATGGTCCTGCTGGAGTTCGTGACCGCCGCCGGGATCAC
TCTCGGCATGGACGAGCTGTACAAGTAA

Cell type classification and clustering. We filtered out 721 cells with less than 100 or more than 3000 genes detected and filtered out 14,388 genes detected in less than 3 cells. We also filtered out 1,066 cells with more than 10% of total counts (UMIs) mapping to mitochondrial genes and 1008 cells determined to be contaminating B cells based on CD19 expression. The raw counts were normalized to 10,000 counts per cell and log(count + 1) transformed. For technical and batch correction, we regressed out total UMI counts and % counts mapping to mitochondrial genes and used combat for batch correction with each sample as a batch. We identified 1119 highly variable genes (excluding all Trav and Trbv genes to avoid clustering cells based on expression of those genes) which were scaled and used with the default settings in scanpy v.1.4.3 [92] for PCA analysis followed by leiden clustering after nearest neighbor detection and uniform manifold approximation and projection

(UMAP) projection. This analysis identified 13 clusters which we collapsed into 9 cell sub-types based on differential gene analysis.

Cell cycle phase assignment and module scoring. To assign cells to the cell cycle phases, the log-normalized scaled gene counts were used with the score_genes_cell_cycle function from the scanpy v.1.5.1 package with the *Mus musculus* G1/S DNA Damage Checkpoints and G2/M Checkpoints gene lists from the REACTOME database being used for the genes associated to the S phase and genes associated to the G2M phase, respectively. For the single cell scoring of the bulk RNA sequencing gene modules, the log-normalized scaled gene counts were used with the score_genes function from scanpy.

RNA velocity analysis. For each 10x well, we used velocyto v.0.17.17 to create a loom file with the spliced, unspliced, and ambiguous counts with the Dec. 2011 GRCm38/mm10 repeat masking gtf file from the UCSC genome browser. The loom files across all wells were merged and then subset to all cells in the T.4NNr4a1 cluster. The resulting object was used to determine the RNA velocity and to predict the latent time for each cell using the 1119 HVGs with the dynamical model from scvelo v.0.2.1.

We used we used a Gaussian mixture model with the GaussianMixture tool from sklearn v.0.23.1 to deconvolute the underlying individual Gaussian distributions from the latent time distribution for cells from the T.4NNr4a1 cluster. This separated the cells into an optimal number of 4 distributions or clusters as determined by the elbow of the Bayesian Information Criterion (BIC) and Akaike Information Criterion (AIC) plots. The smoothed gene expression versus latent time was modelled using a linear generalized additive model using default settings with the LinearGAM function from pygam v.0.8.0. For trajectory inference between the four clusters (“Stage 1” – “Stage 4”), we used the graph-based tool PAGA within scvelo to predict velocity-inferred transitions among the clusters.

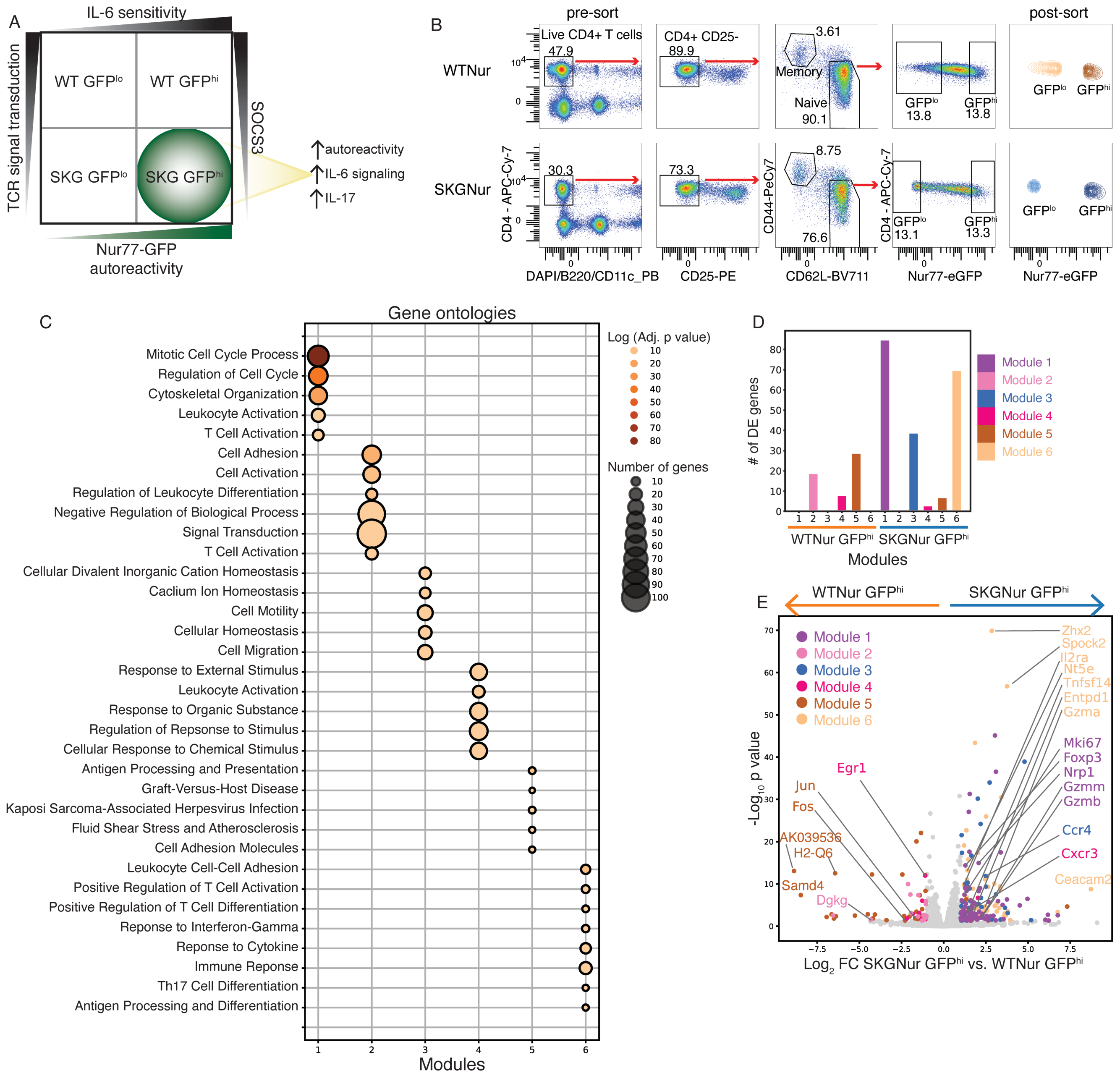

Figure S1. SKGNur GFP^{hi} CD4 T cells readily differentiate into pathogenic effector cells. (**A**) 2x2 matrix demonstrates how impaired TCR signaling observed in SKG mice (left y-axis, due to the hypomorphic *Zap70* allele), in addition to chronic antigen stimulation (x-axis, resulting in higher levels of Nur77-eGFP demarcated by GFP^{hi}) confer heightened sensitivity to IL-6 cytokine signaling, in part due to decreased levels of SOCS3. This contributes to the increased arthritogenicity observed in the autoreactive T cell clones that more readily differentiate into IL-17 producing CD4 T cells in SKG mice. (**B**) Gating for bulk RNAseq sorting of WTNur and SKGNur lymphocytes. (**C**) Dot plot of select pathways from gene ontology analysis for each gene module from **Figure 1C** with dot color indicating adjusted *P* value and dot size proportional to number of genes in overlap between pathway genes and module genes. (**D**) Bar plot of number of DEGs from WTNur GFP^{hi} and SKGNur GFP^{hi} cells contained in each gene module from **Figure 1C**. (**E**) Volcano plot of DEGs for SKGNur GFP^{hi} versus WTNur GFP^{hi}. DEGs are colored by module membership from gene modules in **Figure 1C**.

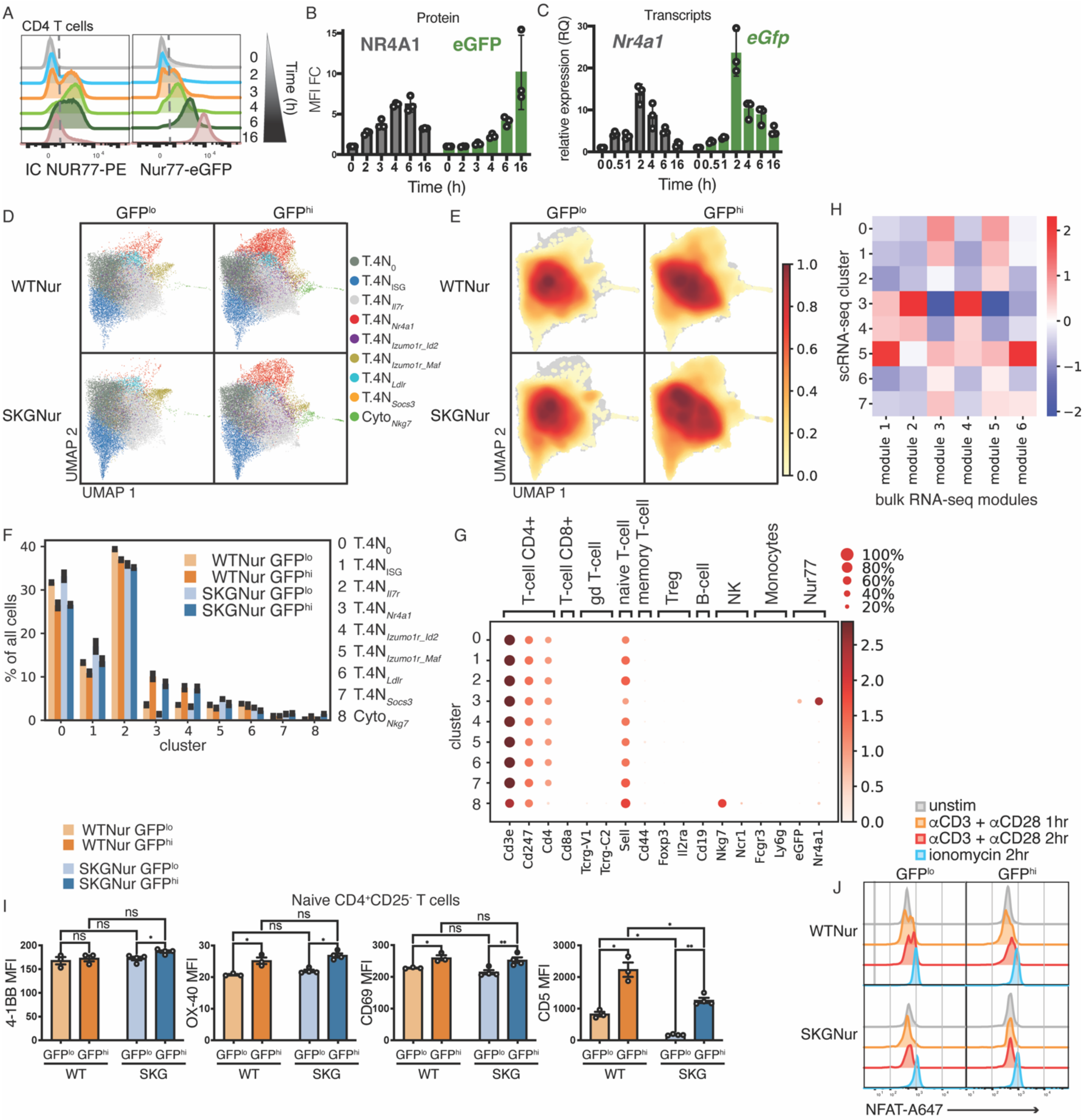

Figure S2. NUR77/*Nr4a1* marks naïve CD4 T cells recently exposed to endogenous antigen, triggering a distinctive transcriptional program. (**A-C**) CD4 T cells were stimulated ± plate bound αCD3ε + soluble αCD28 for the indicated times. Representative histograms in (**A**) show NUR77/NR4A1 and eGFP levels from 2 independent experiments. MFI fold change (FC) quantified in (**B**) from 3 biological replicates. (**C**) Real-time RT-PCR measuring *Nr4a1* and *eGfp* mRNA levels in stimulated CD4 T cells from 3 biological replicates, from 2 independent experiments. (**D-E**) UMAPs colored by merged Leiden clusters (**D**) or by density (**E**) of all cells for each of the four subgroups (each subgroup contains samples from 2 mice). (**F**) Bar plot of mean frequency for each subgroup of cells within each cluster. Black bars indicate difference between mouse 1 and mouse 2 for each subgroup. (**G**) Expression of labelled genes for each cluster is shown by percentage of cells with

expression greater than zero (dot size) and mean expression for cells with nonzero expression (color). (**H**) Heatmap normalized by standard scale (subtract minimum and divide by maximum) by column of average single cell gene set scores for each cluster (excluding cluster 8 – CytoNkg7) for the gene sets defined by the modules from **Figure 1C**. (**I**) Bar graphs show mean fluorescent intensity (MFI ± SEM) of ex vivo expression from indicated surface markers on naïve $CD4^{+}CD25^{-}$ $CD62L^{hi}CD44^{lo}$ T cells from WTNur and SKGNur LN, n = 3-4 mice per genotype, experiment repeated twice. Significance indicated by asterisk [< 0.05 (*), < 0.01 (**), or < 0.001 (***)] for FDR (paired t-test with BH correction) or *P* value (exact permutation test). (**J**) Histograms show NFAT nuclear localization in sorted $CD4^{+}CD25^{-}$ naïve ($CD62L^{hi}CD44^{lo}$) GFP^{hi} and GFP^{lo} cells, stimulated with 5 ug/mL plate-bound αCD3ε + 2 ug/mL soluble αCD28 for 1 or 2 hrs, ionomycin (positive control), or vehicle control from WTNur and 2 pooled SKGNur mice. Data represent 3 independent experiments.

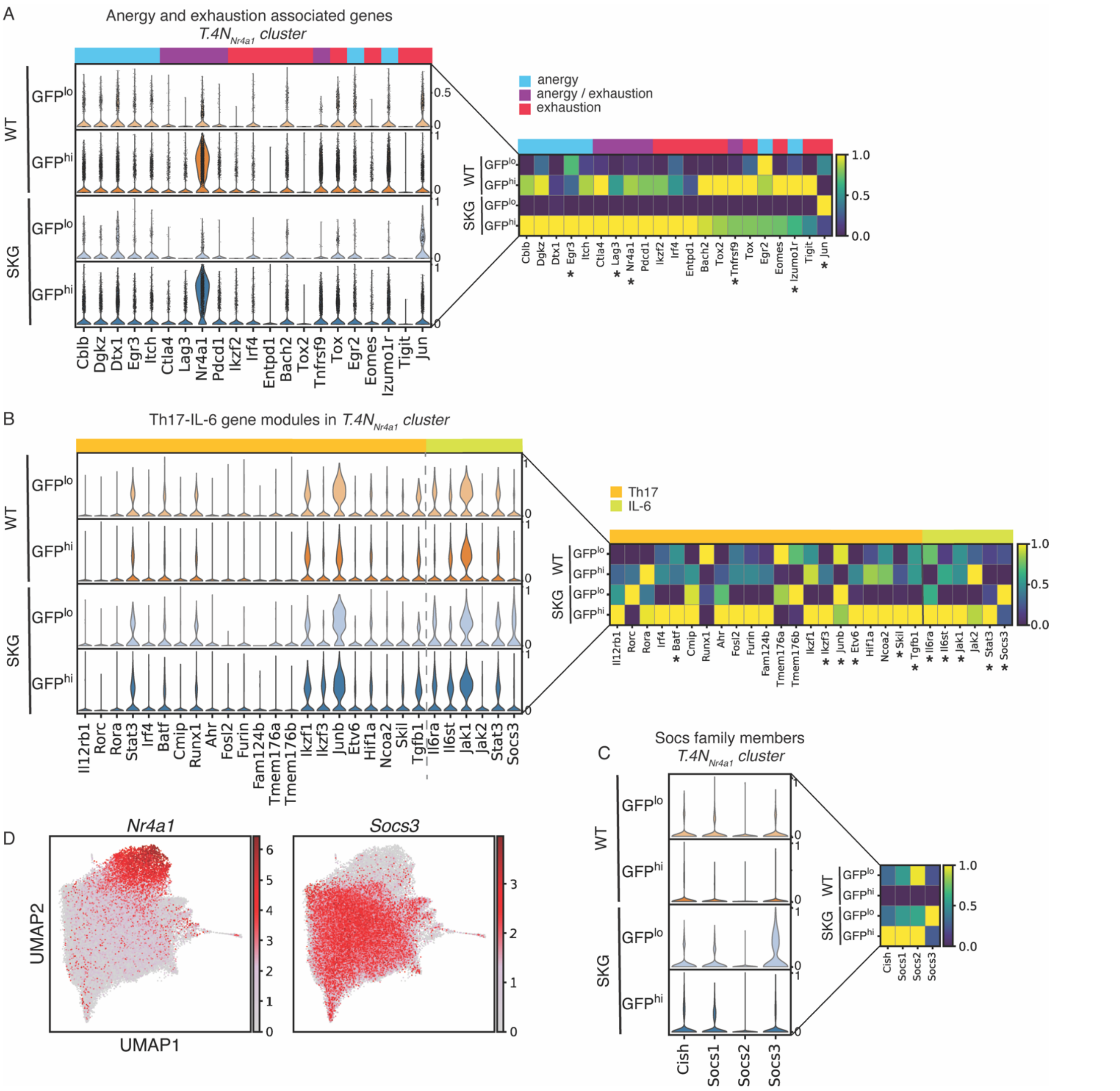

Figure S3. Impaired tolerogenic gene expression and preprogrammed IL-6 hyperresponsiveness in SKGNur arthritogenic T cells. (**A-B**) Stacked violin plot demonstrates standard scale normalized expression of candidate anergy and exhaustion associated genes (**A**) and Th-17 and IL-6 associated genes (**B**) in WTNur and SKGNur GFP^{lo} and GFP^{hi} T.4N$_{Nr4a1}$ cells. Heatmaps on the right for each panel show mean expression of the indicated genes across subgroup normalized by standard scale for each gene. Asterisk (*) next to gene names represented in matrix plots indicates significant DEG between SKGNur GFP^{hi} and WTNur GFP^{hi} cells. *$P < 0.05$, **$P < 0.01$, ***$P < 0.001$, ****$P < 0.0001$. ns, not significant. (**C**) Stacked violin plot demonstrates standard scale normalized expression of *Socs* family members WTNur and SKGNur GFP^{lo} and GFP^{hi} T.4N$_{Nr4a1}$ cells. Heatmap to the right as described in (**A-B**). (**D**) UMAP of all cells colored by expression of the indicated genes. Scale is for the log-normalized gene expression.

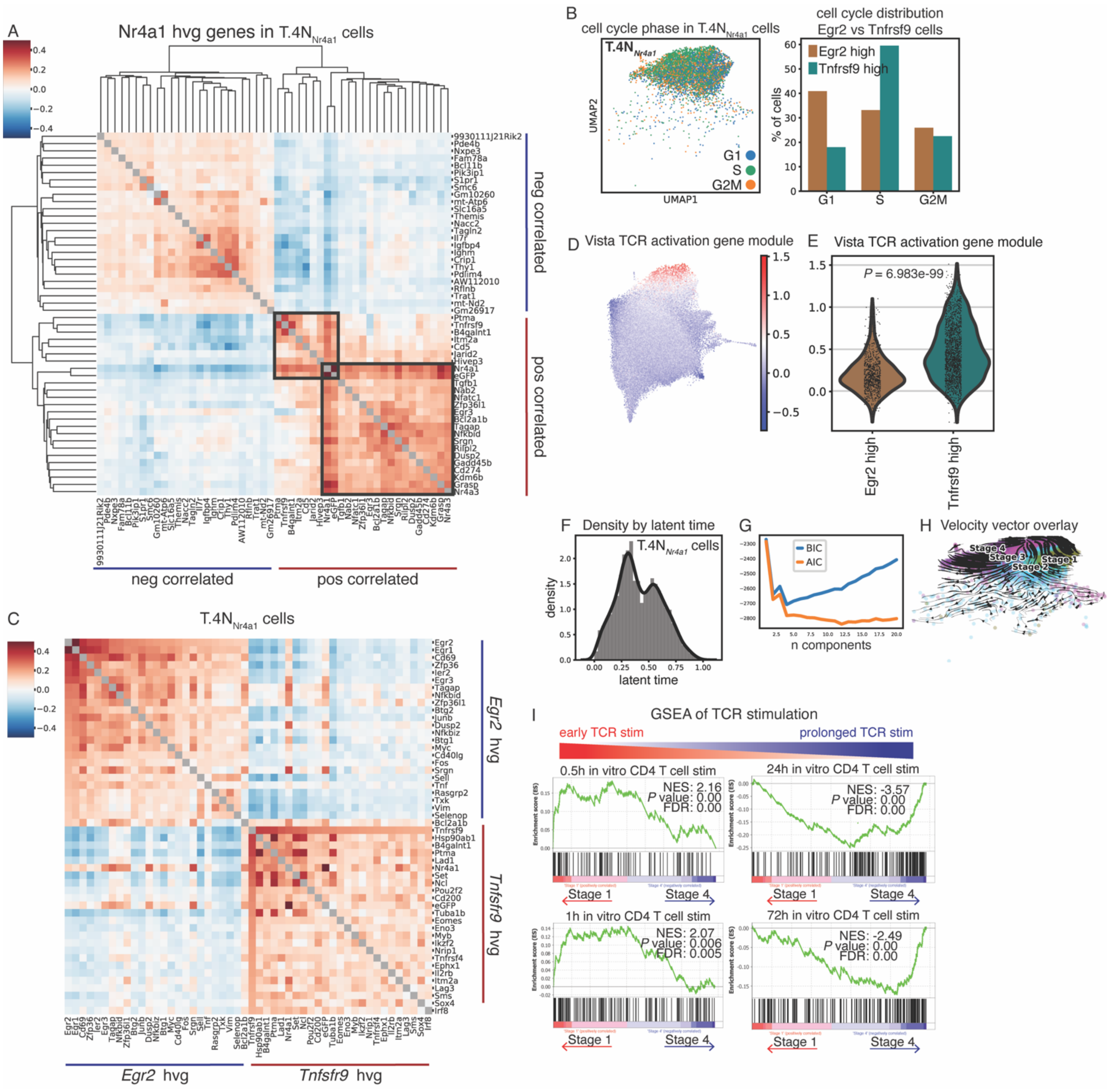

Figure S4. Highly variable genes that positively and negatively correlate with *Nr4a1* in T.4N$_{\mathit{Nr4a1}}$ cluster and trajectory analysis of their underlying states. (**A**) Hierarchical clustering of correlation matrix of top 25 HVGs that positively and negatively correlate with *Nr4a1* expression in WT and SKG T.4N$_{\mathit{Nr4a1}}$ cells using Spearman's correlation. Diagonal grey colored boxes represent correlation of 1. Dark grey boxes mark modules of HVGs that highly correlate with *Nr4a1* expression. (**B**) UMAP of all WT and SKG cells from T.4N$_{\mathit{Nr4a1}}$ cluster colored by cell cycle phase assignment. Bar plot of frequency of cells in each cell cycle stage for cells expressing *Egr2* or *Tnfrsf9* (log-normalized expression > 1). (**C**) Correlation matrix of top 25 HVGs that positively correlate with *Egr2* and *Tnfsrsf9* expression in T.4N$_{\mathit{Nr4a1}}$ cells using Spearman's correlation. Diagonal grey boxes represent correlation of 1. (**D**) UMAP of single cell gene set scores for 'Vista' TCR activation gene module from ElTanbouly et al. (**E**) Violin plot demonstrates single cell gene set scores of 'Vista' TCR activation gene modules

in *Egr2* or *Tnfrsf9* expressing cells from T.4N$_{Nr4a1}$ cluster. Significance indicated by *P* value (linear mixed effect model). (**F**) Probability density of latent time distribution of all cells in T.4N$_{Nr4a1}$ cluster. (**G**) Line plots for the Bayesian Information Criterion (BIC) and Akaike Information Criterion (AIC) for the Gaussian mixture model deconvolution versus number of underlying distributions or clusters. (**H**) UMAP colored by cell stage as defined in **Figure 4D** with an overlay of RNA velocity vectors for cell transitions as determined by the scvelo dynamical model. (**I**) GSEA enrichment plots of pathways in GSE17974 study of CD4$^+$ T cells activated in vitro over time with αCD3ε + αCD28 for ranked genes from DEG analysis of T.4N$_{Nr4a1}$ cluster cells in Stage 1 versus Stage 4. FDR, false discovery rate. NES, normalized enrichment score.

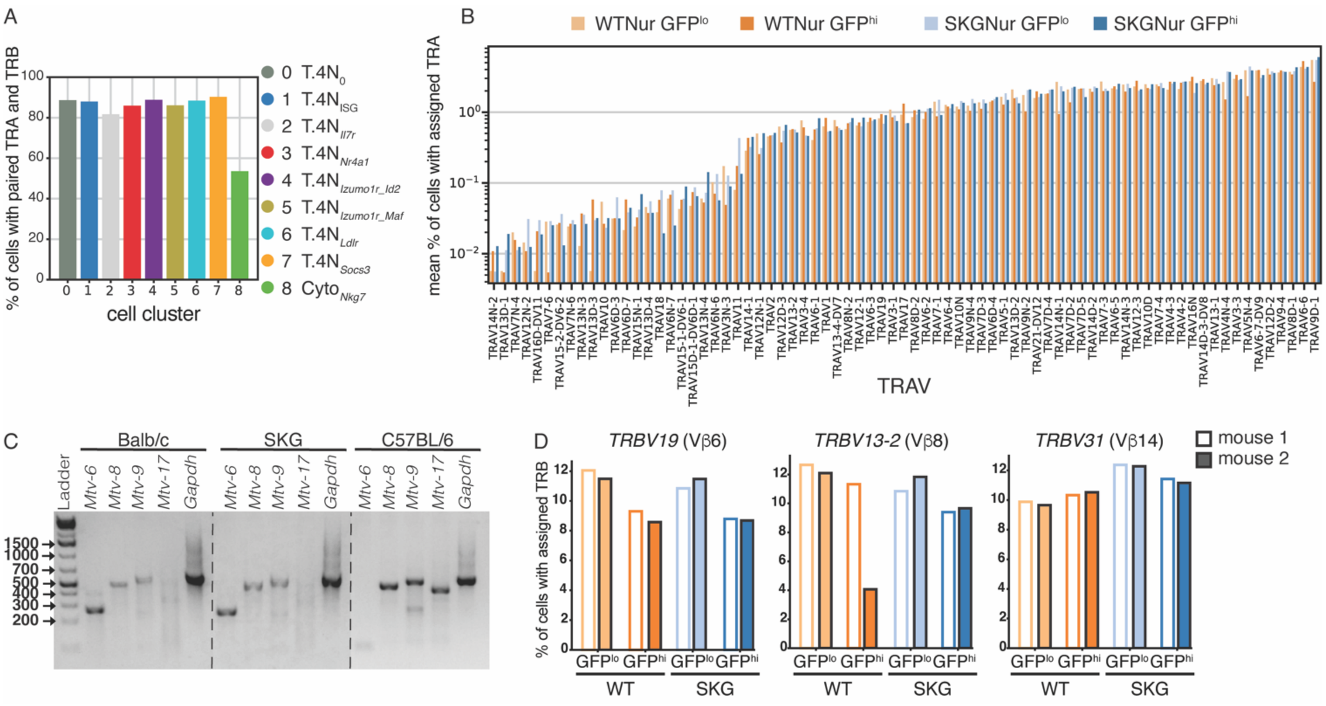

Figure S5. SKGNur mice express superantigens involved in selection of TCR variable beta repertoire. (**A**) Frequency of cells with paired TRA and TRB detected by cluster. (**B**) Mean frequency of cells expressing each TRAV gene as a percentage of all cells in each sample with an assigned TRAV. Bars are colored according to subgroup and ordered by increasing overall frequency. (**C**) BALB/c and SKG tail DNA used in PCR reactions from same experiment containing primers specific for the indicated *Mtv* pro-viruses run on same gel, representative of at least 3-5 biologic replicates per genotype from 3 independent experiments. Dotted lines represent where biological replicates cut out for simplification of data presentation. (**D**) Frequency of cells expressing indicated TRBV control genes not uniquely enriched in SKGNur GFP^{hi} cells for the two replicate mice in each subgroup.

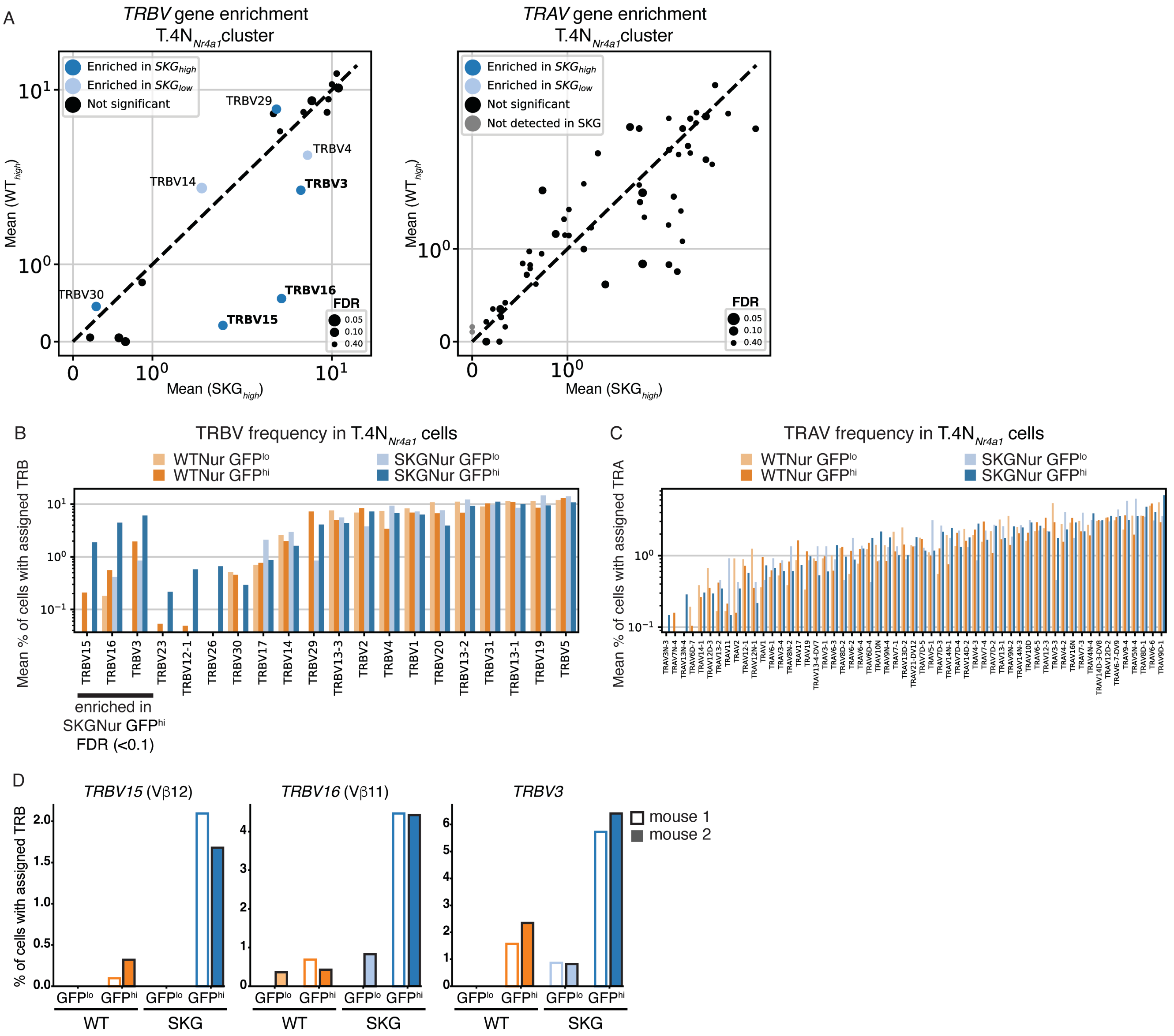

Figure S6. Further enrichment of biased TRBV in SKGNur GFP^{hi} T.4N$_{Nr4a1}$ cells. (**A**) Scatterplot of mean frequency of cells expressing each TRBV (left) or TRAV (right) gene for the SKGNur GFP^{hi} versus the WTNur GFP^{hi} T.4N$_{Nr4a1}$ cells. Dots for each TRV gene are sized according to the false discovery rate (FDR) from a paired one-sided t-test comparing frequency in SKGNur GFP^{hi} versus SKGNur GFP^{lo}. Dots are colored as either significantly enriched (FDR < 0.1) in SKGNur GFP^{hi} (dark blue), significantly enriched in SKGNur GFP^{lo} (light blue), or not significantly enriched in either subgroup (black). TRBV genes that were significantly and uniquely enriched in SKGNur GFP^{hi} cells within T.4N$_{Nr4a1}$ cluster are bolded. (**B**) Bar plot of mean value of T.4N$_{Nr4a1}$ cells expressing each TRBV gene as a percentage of all T.4N$_{Nr4a1}$ cells in each sample with an assigned TRBV. Bars are colored according to subgroup and are ordered with the TRBV genes enriched in SKGNur GFP^{hi} T.4N$_{Nr4a1}$ cells (see **figure S6A**) followed by the other TRBV genes ordered by increasing overall frequency. (**C**) Bar plot of mean value of T.4N$_{Nr4a1}$ cells expressing each TRAV gene as a percentage of all T.4N$_{Nr4a1}$ cells in each sample with an assigned TRAV. Bars are colored according to subgroup and are ordered by increasing overall frequency. (**D**) Frequency of cells expressing indicated TRBV genes significantly enriched in SKGNur GFP^{hi} T.4N$_{Nr4a1}$ cells (see **figure S6A**) for two replicate mice in each subgroup.

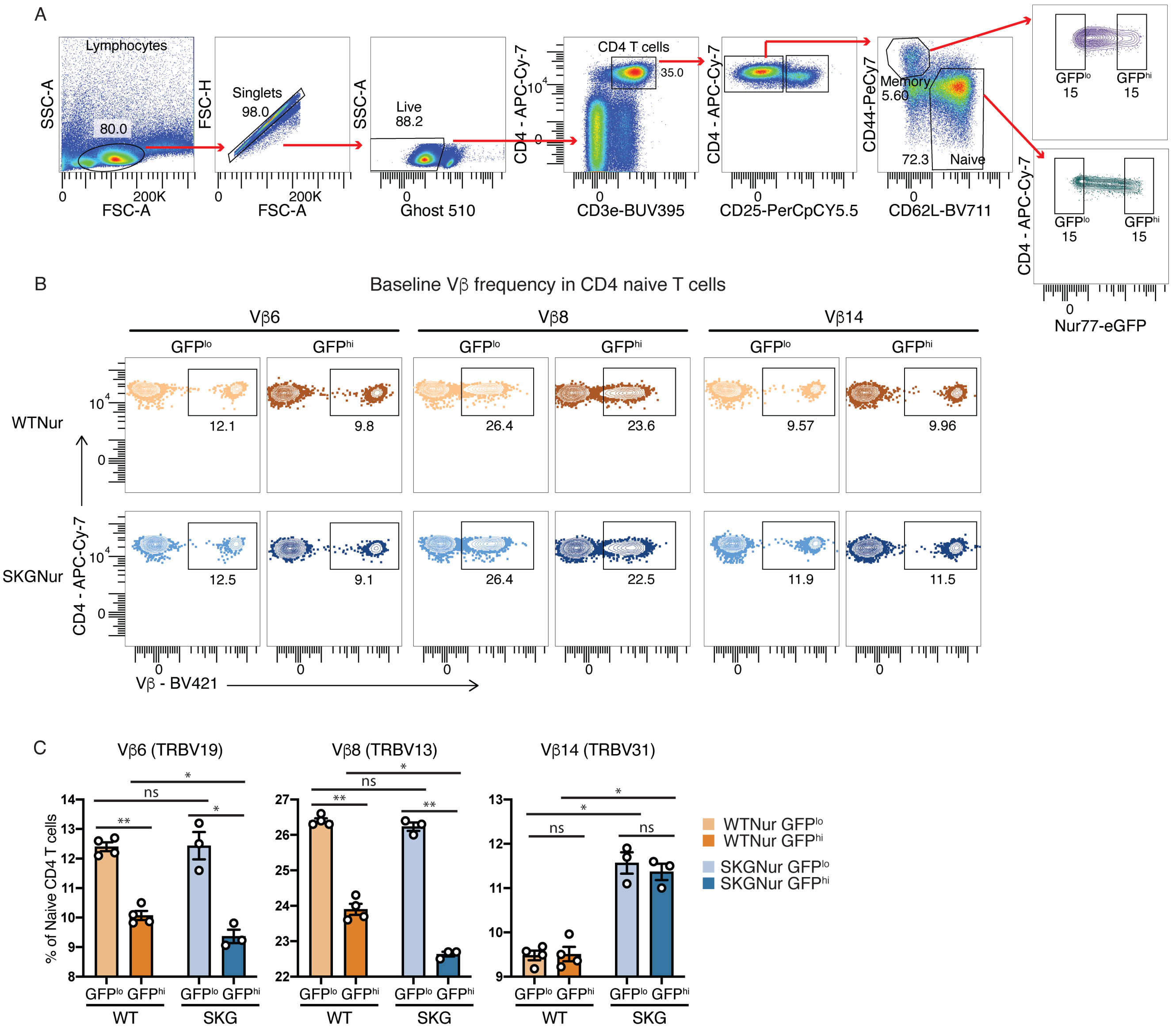

Figure S7. TCR Vβs unresponsive to BALB/c MMTV superantigen do not expand in antigen activated GFP^{hi} T cells. (**A**) Flow cytometry gating used to identify GFP^{hi} and GFP^{lo} populations in naïve (CD62L^{hi}CD44l^{o}) and memory (CD44^{hi}CD62L^{lo}) CD4^{+}CD25^{-} T cells for Vβ identification in WTNur and SKGNur lymphocytes. (**B-C**) Representative FACS plots in (**B**) of naïve peripheral CD4 T cells with indicated TCR Vβ protein usage determined by flow cytometry in GFP^{lo} and GFP^{hi} T cells from LN of WTNur and SKGNur mice prior to arthritis induction and quantified in (**C**) where bar graphs depict mean frequency (± SEM), n = 3-4 mice per group, experiment repeated at least 3 times. Significance indicated by asterisk for FDR (paired t-test with Benjamini-Hochberg (BH) correction) or *P* value (exact permutation test) < 0.05 (*), < 0.01 (**), or < 0.001 (***).

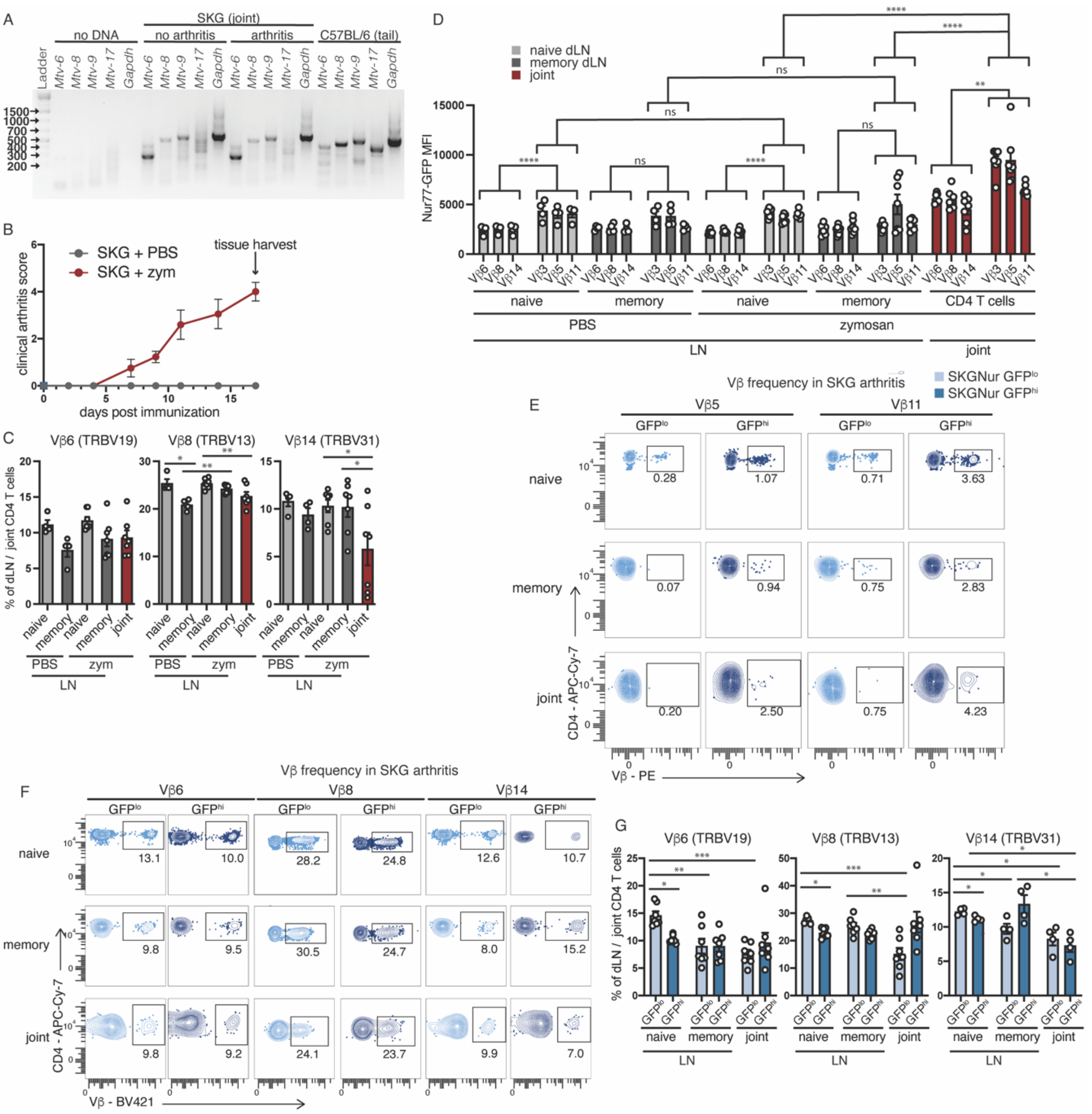

Figure S8. TCR Vβs unresponsive to BALB/c MMTV superantigen do not expand in SKG CD4+ T cells after arthritis induction. (**A**) DNA was used from SKG joints ± arthritis in PCR reactions containing primers specific for the indicated *Mtv* pro-viruses. Lanes 2-6 show PCR mixtures lacking template DNA. C57BL/6 tail DNA was used as a positive control for *Mtv-8, -9,* -17 and a negative control for *Mtv*-6. Molecular size markers are shown in lane 1. Each gel is representative of at least 3-4 biological replicates per condition and genotype. (**B**) Arthritis score in SKGNur mice ± i.p. zymosan (red) or PBS (grey), n=4 mice in each group, representative of at least 3 experiments. (**C**) Bar graph depicts mean frequency (± SEM) of peripheral naïve or memory, or joint CD4 T cells with indicated TCR Vβ protein usage determined by flow cytometry in CD4 T cells from draining LN or joints of SKGNur mice 2.5 weeks after arthritis induction with zymosan, as seen in (**B**), n = 7 mice per group pooled from 2 experiments. (**D**) Bar graphs of Nur77-eGFP mean fluorescence intensity (MFI ± SEM) of $CD4^+CD25^-$ naïve ($CD62L^{hi}CD44^{lo}$) and memory ($CD44^{hi}CD62L^{lo}$) cells from SKG LN after zymosan treatment (n = 7) or PBS vehicle control (n = 4) or $CD4^+$ T cells from SKG arthritic joints after zymosan treatment expressing indicated Vβs (n = 7), pooled from 2 experiments. (**E-G**)

Representative FACS plots of peripheral naïve or memory, or joint CD4 T cells with indicated TCR Vβ protein usage determined by flow cytometry in GFP^{lo} (light blue) and GFP^{hi} (dark blue) T cells from LN or joints of SKGNur mice 2.5 weeks after arthritis induction with zymosan and quantified in (**G**) or **Figure 6E** where bar graphs depict mean frequency (± SEM), n = 7 mice per group pooled from 2 experiments or n = 4 mice (for Vβ14). Significance indicated by asterisk [< 0.05 (*), < 0.01 (**), or < 0.001 (***)] for FDR (paired t-test with BH correction) or *P* value (exact permutation test) (**C** and **G**) or adjusted *P* value (linear mixed effect model with BH correction) (**D**).

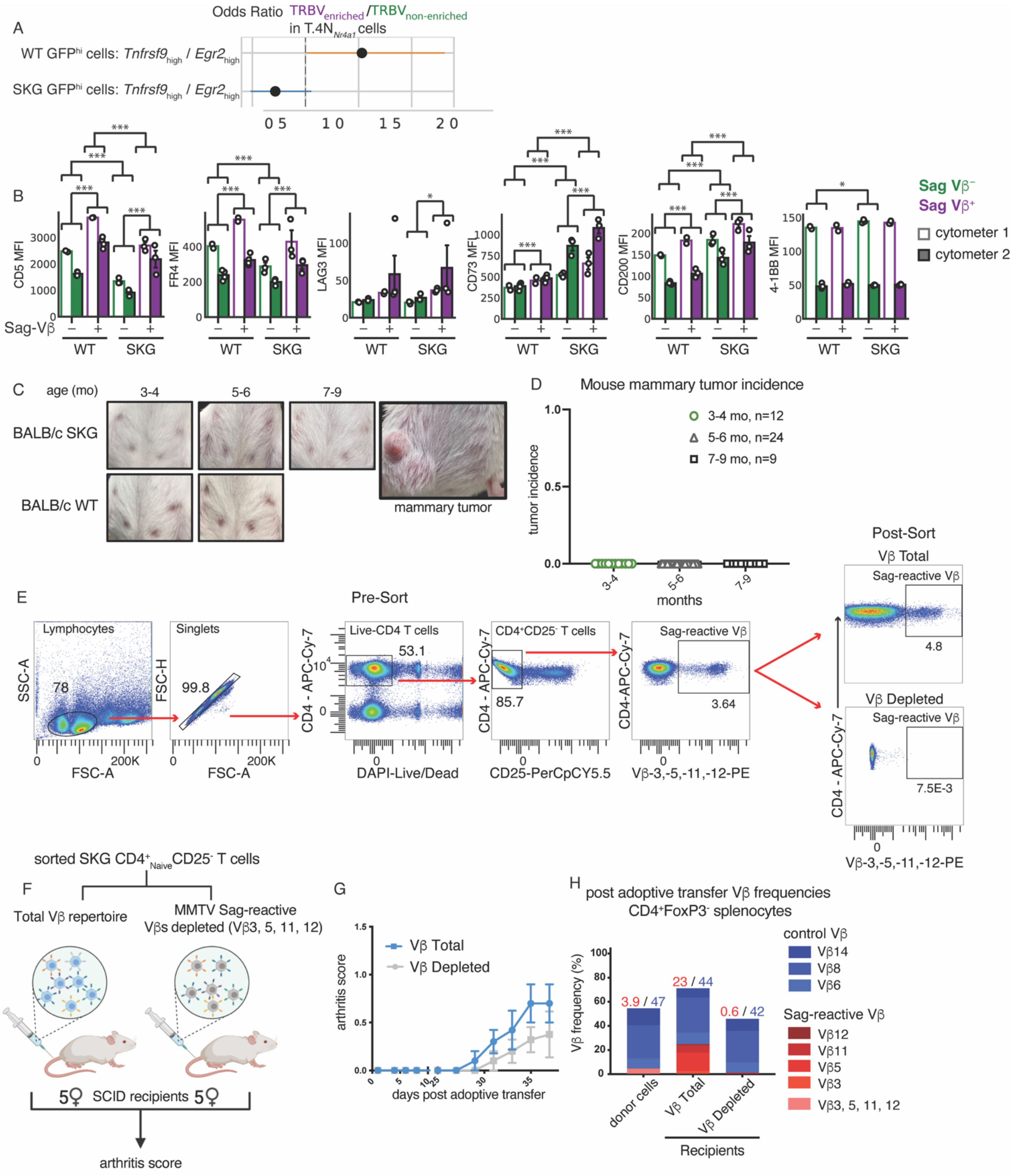

Figure S9. Sag-reactive SKG T cells harbor arthritogenic cells. (**A**) Line plot of odds ratio (± 95% CI) that cells with an assigned TRBV which is part of the enriched TRBVs (*TRBV3*, *12-1*,*15*, *16*, and *29*) rather than the non-enriched TRBVs are in the *Tnfrsf9* subcluster versus the *Egr2* subcluster for

WT GFP^{hi} cells and SKG GFP^{hi} cells. (**B**) Bar graphs represent quantification of ex vivo protein expression of indicated markers in $CD4^+CD25^-$ naïve Sag-reactive ($V\beta3^+V\beta5^+V\beta11^+V\beta12^+$) or Sag non-reactive ($V\beta3^-V\beta5^-V\beta11^-V\beta12^-$) T cells from LNs of n=6 mice per genotype from 4 independent experiments. Significance indicated by asterisk [< 0.05 (*), < 0.01 (**), or < 0.001 (***)] for adjusted *P* value (linear mixed effect model with BH correction). (**C-D**) Mammary glands from 3–9-month-old SKG female mice were palpated to examine for tumors. (**C**) Representative photos of mouse mammary glands and a sample tumor (right panel). (**D**) Quantification of mouse mammary tumor incidence in SKG mice at 3-4 months (n=12), 5-6 months (n=24), 7-9 months (n=9). (**E**) Flow cytometry gating and sorting strategy used for **Figure 8, E-H**. (**F**) Sorted SKG $CD4^+CD25^-$ naïve T cells of the indicated Vβ T cell populations were adoptively transferred into SCID recipients and monitored for arthritis development. (**G**) Arthritis score in SCID mice after adoptive transfer in (**F**). (**H**) Stacked bar graph depicting mean frequency of indicated Vβs from SKG donor cells (combined Sag-reactive Vβs) and in SCID recipient splenocytes 5 weeks after adoptive transfer from experiment in (**F**), data pooled from 5 mice in each group.

Genotype	H-2 haplotype	Provirus	Vβ Specificity	Size (bp)
BALB/c	*d*	*Mtv-6*	3, 7	265
		Mtv-8	11, 12	488
		Mtv-9	5, 11, 12	537
C57BL/6	*b*	*Mtv-8*	11, 12	488
		Mtv-9	5, 11, 12	537
		Mtv-17	?	434

Table S1. MMTV proviruses and Vβ specificity in BALB/c and C57BL/6 mice. H-2 haplotype, expected Mtv pro-virus and its Vβ specificity, and base pair (bp) size on gel for BALB/c and C57BL/6 mice.

Supplementary Data

Data S1. (data_S1_bulk_RNA_seq_diff_exp.xlsx)
Differential expression results for all bulk RNA seq comparison.

Data S2. (data_S2_heatmap_gene_list_with_modules.xlsx)
List of genes in heatmap in **Figure 1C**.

Data S3. (data_S3_GSEA_reports.xls)
GSEA reports for all GSEA analyses.

Data S4. (data_S4_scRNAseq_diff_genes_by_cluster.xlsx)
Differential expression results for single cell RNA sequencing clusters.

Data S5. (data_S5_fig_2_diff_gene_lists.xlsx)
Differential expression results for analyses in **Figure 2**.

Data S6. (data_S6_fig_3_diff_exp_lists.xlsx)
Differential expression results for analyses in **Figure 3**.

Data S7. (data_S7_top_300_heatmap_gene_list.tsv)
List of genes in heatmap in **Figure 4F**.

Data S8. (data_S8_gini_coefficients.xlsx)
Gini coefficients for clonotype distribution for cells in each cluster from each mouse.

Data S9. (data_S9_diff_exp_TRBV_enriched_TRBV_non_enriched.xlsx)
Differential expression results for analyses in **Figure 7**.
