# Supplementary figures and images for: Endogenous antigens shape the transcriptome and TCR repertoire in an autoimmune arthritis model
Source: J Clin Invest. 2024 Nov 26;135(2):e174647. doi: 10.1172/JCI174647 (PMC11735108; doi:10.1172/JCI174647)

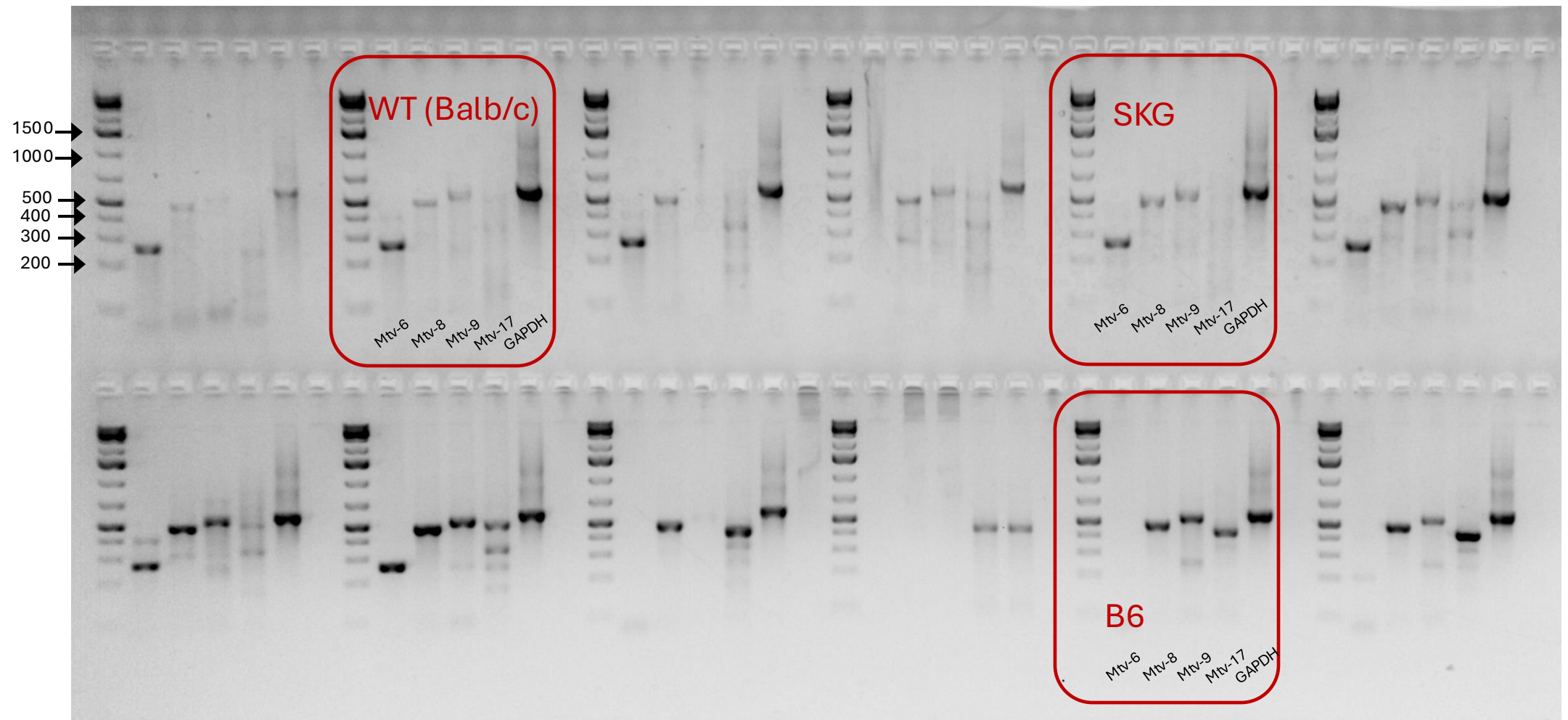

Full unedited gel for supplemental figure S8A

Supplement: Unedited blot and gel images [file jci-135-174647-s224.pdf]
